# Sliding Ferroelectric Tunnel Junctions with Ultrahigh Tunneling Electroresistance

**DOI:** 10.1021/acsnano.6c11972

**Published:** 2026-10-20

**Authors:** Haolin You, Chen Shao, Saagar Kolachina, Adrian Jonathan Lim, Robert Boyd, Chengdong Wang, Fan Fei, Carter Fox, Sina Najmaei, Jun Xiao, Daniel A. Rhodes, Xiaofeng Qian, Ying Wang

**Affiliations:** † Department of Electrical and Computer Engineering, 5228 University of Wisconsin−Madison, 1415 Engineering Dr., Madison, Wisconsin 53706, United States; ‡ Department of Materials Science and Engineering, 14736 Texas A&M University, 575 Ross St., College Station, Texas 77843, United States; § Department of Materials Science and Engineering, University of Wisconsin−Madison, 1509 University Ave., Madison, Wisconsin 53706, United States; ∥ Department of Physics, University of Wisconsin−Madison, 1150 University Ave., Madison, Wisconsin 53706, United States; ⊥ DEVCOM Army Research Laboratory, Adelphi Laboratory Center, Adelphi, Maryland 20783, United States; # Department of Physics and Astronomy, Texas A&M University, 578 University Dr., College Station, Texas 77843, United States; ∇ Department of Electrical and Computer Engineering, 14736 Texas A&M University, 188 Bizzell St., College Station, Texas 77843, United States

**Keywords:** sliding ferroelectricity, ferroelectric tunnel junctions, 3R-MoS_2_, van der Waals materials, interfacial electrostatics, strain engineering, nonvolatile memory

## Abstract

Sliding ferroelectricity in van der Waals materials offers an appealing route to compact, nonvolatile, atomically thin ferroelectric tunnel junctions (FTJs) with low switching energy, but achieving a large resistance contrast in such devices remains challenging because the weak out-of-plane polarization provides limited electrostatic modulation of the tunneling barrier. Here, using bilayer rhombohedral MoS_2_ as a sliding ferroelectric barrier, we demonstrate room-temperature sliding FTJs with tunneling electroresistance (TER) values exceeding 3.2 × 10^5^. We show that this giant contrast arises from a height–width hybrid tunneling mechanism, in which polarization reversal simultaneously modulates the tunnel barrier height and switches a nanoscale interfacial depletion width, yielding exponential amplification of the tunneling contrast. Spatially resolved tunneling, piezoresponse, and Raman measurements reveal that local strain, rather than ferroelectric domain walls, dominates spatial variations in the TER by modulating the sliding ferroelectric dipole strength, the OFF-state depletion width, and the resulting TER amplification. Finally, planar 3R-MoS_2_ FTJs further validate this mechanism by exhibiting a TER above 10^5^, a switching energy density of several fJ μm^–2^, and projected endurance beyond 10^6^. These results provide fundamental insight into the nanoscale electrostatics in sliding ferroelectric-based tunnel junctions while pointing toward low-energy, high-contrast nonvolatile memory applications.

## Graphical abstract

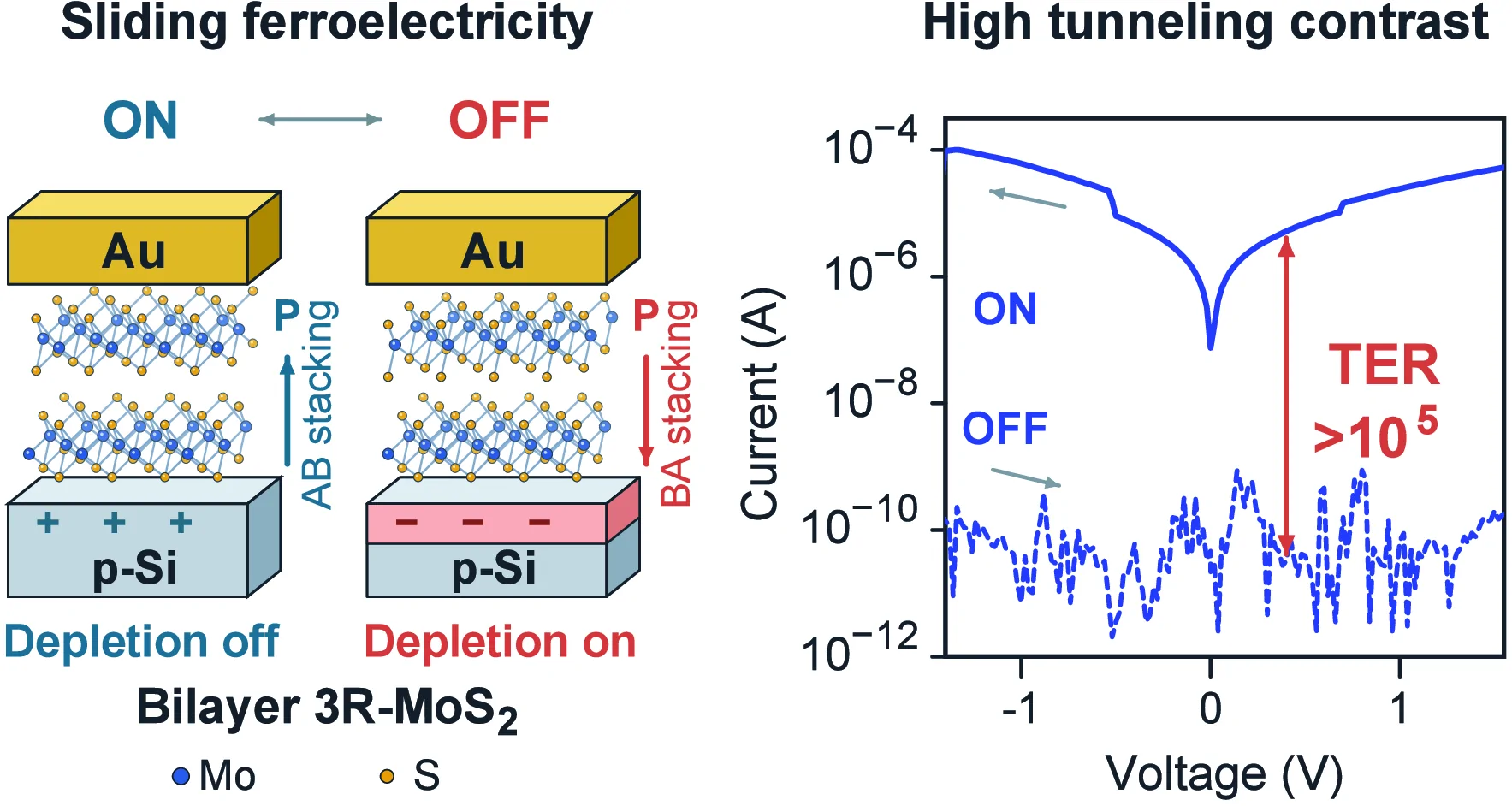

The rapid growth of artificial intelligence, edge computing, and data-intensive electronics has created an urgent need for energy-efficient memory devices that can colocate computation and storage, thereby reducing the latency and energy cost associated with the von Neumann bottleneck. Neuromorphic and in-memory computing architectures are particularly reliant on dense crossbar arrays of two-terminal, nonvolatile memory elements that emulate synaptic weights with analog tunability, low power consumption, and high endurance.^1^ Among the various ferroelectric memories, ferroelectric tunnel junctions (FTJs) are especially attractive because their simple electrode/ferroelectric/electrode geometry directly converts polarization reversal into a nonvolatile tunneling resistance contrast. This compact two-terminal architecture enables nondestructive readout, low standby power, high integration density, and strong scaling potential.^2–5^ These features distinguish FTJs from other ferroelectric memory technologies, which suffer from destructive readout,^6^ short-channel effects, gate-stack scaling, and limited array density.^7,8^ However, achieving FTJs that simultaneously combine large tunneling electroresistance, low switching energy, robust endurance, and aggressive thickness scaling remains challenging because only a limited class of ferroelectric barriers can satisfy these competing requirements.

Sliding ferroelectricity has recently emerged as a distinct form of ferroelectric order in van der Waals (vdW) materials, where polarization reversal occurs through interlayer sliding rather than ionic displacement.^9–12^ It has been observed both in exfoliated crystals,^13–17^ such as 3R-MoS_2_ and T_d_-WTe_2_, and in artificially assembled heterostructures, including twisted boron nitride (BN)^18,19^ and transition metal dichalcogenides (TMDs),^20^ where tailored stacking sequences induce robust out-of-plane polarizations. Because adjacent layers are bound by weak van der Waals interactions, sliding between opposite polarization states can proceed through low-energy stacking transitions,^12^ enabling low switching voltages, fast operation, and excellent endurance.^21–23^ These properties make sliding ferroelectrics appealing candidates for ultrathin, low-power FTJs. Despite this promise, high-performance sliding FTJs have remained difficult to realize because the intrinsically weak out-of-plane polarization of sliding ferroelectrics, typically in the sub-μC cm^–2^ range, is 1 to 2 orders of magnitude smaller than that of conventional ferroelectrics such as BaTiO_3_ and HfO_2_.^8,24^ Such weak polarization provides only limited electrostatic modulation of the tunneling barrier. As a result, theoretical studies predict a tunneling electroresistance (TER), the ratio between high- and low-resistance states that quantifies the electrical contrast of the junction, typically on the order of 10^2^ or below, while FTJs on sliding ferroelectric systems such as twisted boron nitride and twisted bilayer MoS_2_ show TER values below 10.^25,26^ This creates an apparent paradox: sliding ferroelectrics offer the desired ultrathin, low-energy, high-endurance switching medium, yet their small polarization appears insufficient to generate large tunneling electroresistance through barrier modulation.

Here, we demonstrate that this weak polarization limitation can be overcome by a height–width hybrid tunneling mechanism. Using bilayer rhombohedral (3R) MoS_2_ as the sliding ferroelectric barrier, we realize room-temperature sliding FTJs with an ultrahigh TER exceeding 3.2 × 10^5^. We show that the sliding polarization acts as a nanoscale electrostatic trigger that modulates not only the tunnel barrier height but also an interfacial depletion width at depletion-capable contacts. This coupled height–width modulation exponentially amplifies the tunneling contrast beyond what is achievable from barrier height modulation alone.

We validate this mechanism through contact-dependent junctions, spatially resolved measurements, and planar two-terminal devices. Depletion-capable p-Si and boron-doped diamond contacts produce giant TER values above 10^5^, with scanning capacitance microscopy confirming depletion-type interfacial behavior, whereas Pt and few-layer graphene control junctions show TER values below 10, ruling out barrier height modulation alone as the origin of the giant TER. Multiple planar Au/3R-MoS_2_/p-Si devices reproduce TER values above 10^5^ while maintaining a low switching energy density of 4.31 fJ μm^–2^ and projected endurance beyond 10^6^ cycles.

The combination of spatially resolved tunneling, piezoresponse, and Raman measurements further identifies tensile strain rather than ferroelectric domain walls as the microscopic origin of spatial TER variation: strain locally modifies the sliding polarization, and it strongly narrows the depletion width of the OFF state, increasing the OFF-state tunneling current and weakening the depletion-amplified resistance contrast. This provides a design guideline for preserving uniform, high-contrast switching in sliding FTJs. Together, these results establish height–width hybrid tunneling as a mechanism for converting weak sliding ferroelectric polarization into a large resistance contrast and provide a route toward ultrathin, low-energy, high-contrast nonvolatile memory devices.

## Results

We first show the device geometry and electric field switchability of sliding ferroelectricity, which are essential for realizing sliding ferroelectric tunnel junctions. As illustrated in Figure 1a, the FTJ consists of a bilayer 3R-MoS_2_ flake sandwiched between two asymmetric contacts. Bilayer 3R-MoS_2_ is selected as the ferroelectric layer because its rhombohedral stacking breaks inversion symmetry and produces an interlayer dipole with a well-defined out-of-plane polarization.^14,27,28^ In the bilayer limit, the polarization is determined by the relative layer registry, giving two sliding ferroelectric states, denoted AB and BA stacking (Figure 1a).^14,27,28^ In the AB configuration, Mo atoms in the upper layer align above S atoms in the lower layer, whereas this registry is reversed in the BA configuration, resulting in opposite polarization directions that potentially modulate the tunneling barrier. Contact mode AFM confirms the uniform thickness and surface morphology of those bilayer flakes in FTJs (Figure 1b and Supplementary Note 1). We use two top-electrode configurations: a tip-based Au/3R-MoS_2_/boron-doped diamond (conducting BDD tip) geometry for nanoscale, spatially resolved tunneling measurements and a lithographically defined Au/3R-MoS_2_/p-Si geometry for planar two-terminal device characterization (see Methods).

**Figure 1:**
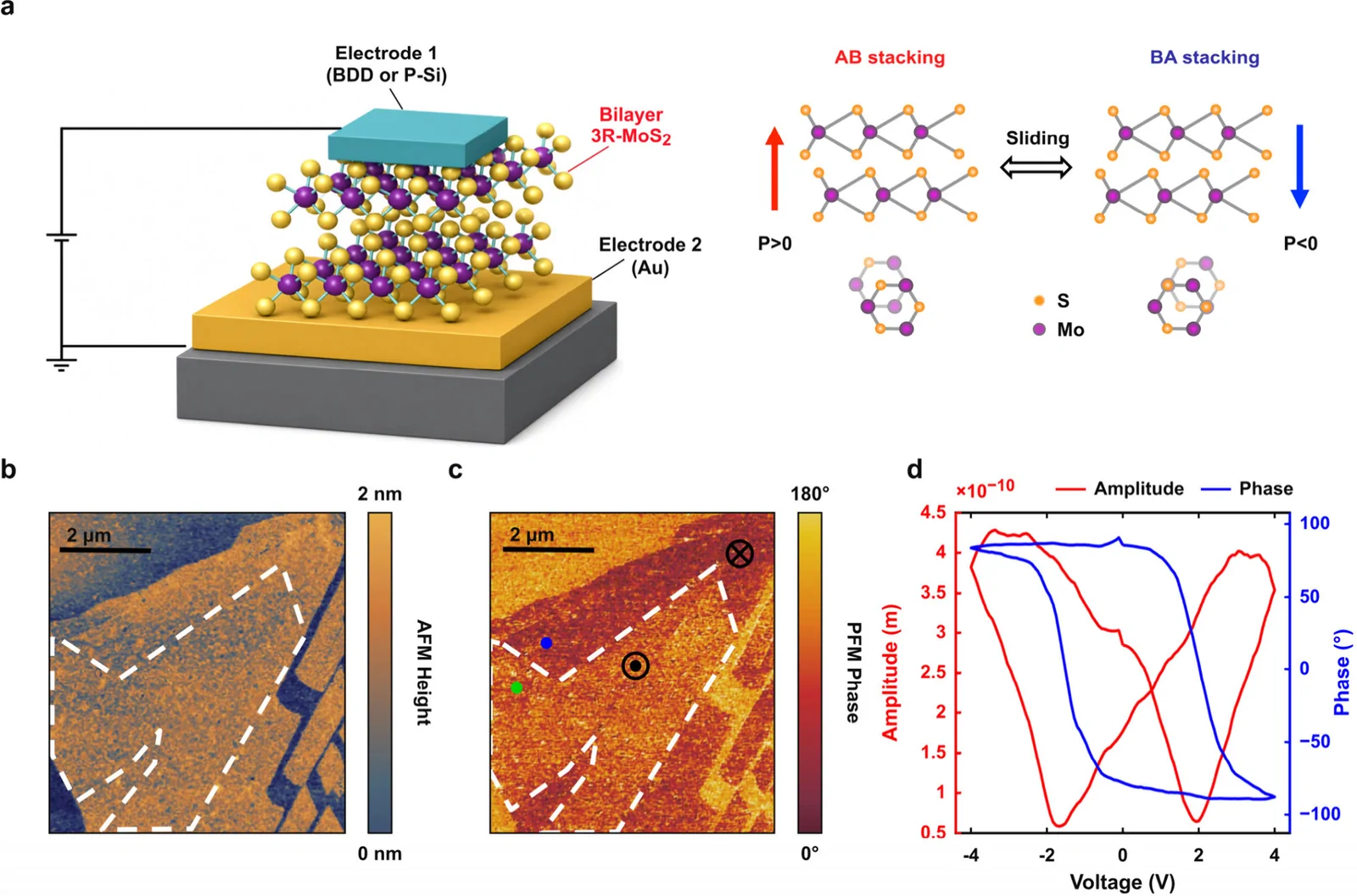
Device structure and ferroelectric switching of bilayer 3R-MoS_2_ for sliding FTJs. **a**, Left: Schematic of the sliding FTJ consisting of a bilayer 3R-MoS_2_ flake sandwiched by two asymmetric electrodes. The junction is implemented using either a nanoscale BDD AFM tip together with an Au contact or a lithographically defined doped-silicon substrate and Au electrode in planar devices. Right: AB and BA stacking configurations in bilayer 3R-MoS_2_, which have opposite out-of-plane polarizations arising from layer registry. **b**, AFM topography of a representative bilayer 3R-MoS_2_ flake, confirming the uniform thickness and surface morphology. **c**, Vertical PFM phase image, revealing distinct out-of-plane ferroelectric domains separated by white dashed lines, with upward and downward polarizations for AB and BA domains marked by ⊙ and ⊗, respectively. Scale bars: 2 μm. **d**, Out-of-plane amplitude and phase response of 3R-MoS_2_ measured by SS-PFM, confirming electric-field-induced polarization switching.

To verify the ferroelectric domain structure and switchability of the bilayer 3R-MoS_2_, vertical piezoresponse force microscopy (PFM) is used, and it reveals two domains with 180° phase contrast,^20,29,30^ assigned to opposite out-of-plane polarization states associated with AB and BA stacking domains (Figure 1c). Reversible electric field switching was confirmed by switching spectroscopy PFM (SS-PFM), in which the remanent piezoresponse was measured after DC switching pulses (see Methods). The butterfly shaped amplitude loop and hysteretic phase reversal confirm polarization switching between the two sliding ferroelectric states (Figure 1d). Together, these measurements establish that bilayer 3R-MoS_2_ provides electrically switchable, nonvolatile polarization states suitable for FTJ operation. The polarization directions are further corroborated by Kelvin probe force microscopy (KPFM) measurements (Supplementary Note 2).

We next evaluate whether the sliding polarization in bilayer 3R-MoS_2_ can generate a large tunneling resistance contrast. Using a tip-based vertical Au/3R-MoS_2_/boron-doped diamond (BDD) FTJ geometry, in which a conductive BDD AFM tip serves as the movable top electrode, we performed conductive atomic force microscopy (CAFM) measurements on individual AB and BA ferroelectric domains. Representative *I*–*V* curves measured at P1 and P2, corresponding to BA and AB domains, respectively, show clear hysteresis associated with reversible polarization-controlled switching between ON and OFF tunneling states (Figure 2a,b). Repeated voltage sweeps at the same location yield nearly identical hysteresis loops, indicating stable and reproducible switching of the local tunneling conductance. TER, extracted from *I*_ON_/*I*_OFF_ at the same read bias, exceeds 10^5^.

**Figure 2:**
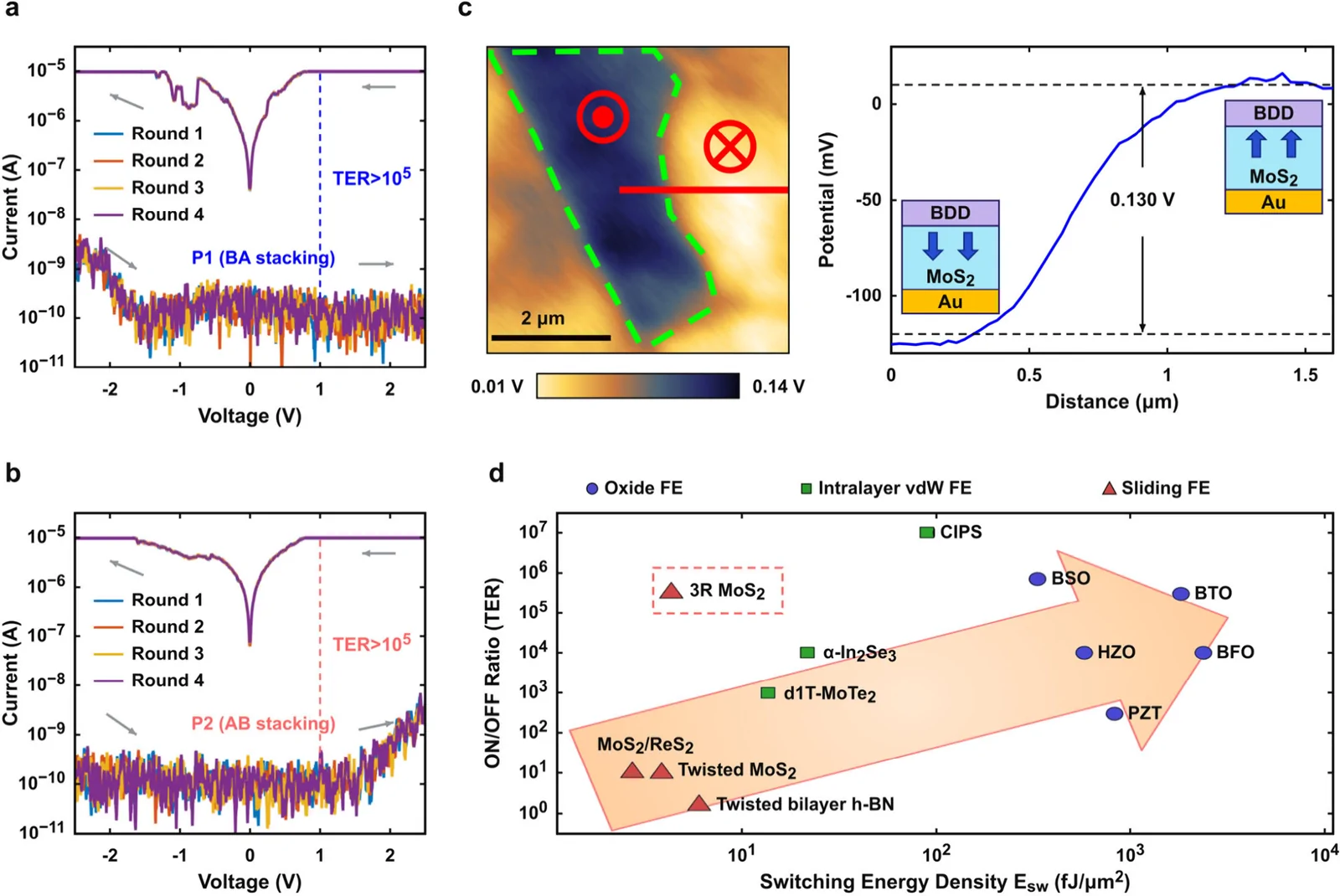
Giant tunneling electroresistance despite weak polarization of sliding ferroelectricity. **a**,**b**, Representative *I*–*V* curves measured at P1 and P2, corresponding to positions indicated by the blue (BA stacking) and green (AB stacking) points in Figure 1c, showing reproducible hysteretic tunneling and TER over 10^5^. Gray arrows indicate the voltage sweep direction. **c**, KPFM surface potential map of the bilayer 3R-MoS_2_ on Au and corresponding line profile acquired along the marked red line across two adjacent polarization domains. Polarization directions are marked by red markers (⊙ for polarization pointing out of the plane in AB stacking, and ⊗ for polarization pointing into the plane in BA stacking) and domains separated by green dashed lines. The line profile reveals a surface potential step of ∼0.130 V between opposite polarizations, revealing the weak electrostatic contrast associated with sliding ferroelectricity. Insets reveal the corresponding polarization orientations and device stacks. **d**, Benchmarking of TER versus the dipole switching energy density for reported FTJs based on ferroelectric oxides (circles),^34–36,41,42^ intralayer van der Waals ferroelectrics (squares),^38,39^ as well as sliding ferroelectrics (triangles).^37,43,44^ The red dashed box highlights the performance of the Au/bilayer 3R-MoS_2_/BDD sliding FTJ, which combines ultrahigh TER and ultralow switching energy density. The orange arrow is a guide to the eye indicating the conventional trend in which large TER typically requires large switching energy densities; the present sliding FTJ deviates from this trend by achieving a giant TER despite the weak polarization of bilayer 3R-MoS_2_.

To quantify the polarization of bilayer 3R-MoS_2_ that contributes to the switching in such FTJs, we performed KPFM across adjacent AB and BA domains with opposite out-of-plane polarizations. The opposite polarization states generate different bound surface charges, giving rise to a measurable surface potential step between domains.^31^ The KPFM map and line profile resolve a potential difference of approximately 0.13 V across the domain boundary (Figure 2c). Treating the ferroelectric bilayer 3R-MoS_2_ as a parallel-plate capacitor, this potential step provides an estimate of the remanent polarization, *P* = (ε_0_ε_r_Δ*V*_KPFM_)/2*d*, where Δ*V*_KPFM_ is the measured potential difference, *d* is the thickness of the ferroelectric layer, *ε*_0_ represents the vacuum permittivity, and ε_r_ is its relative permittivity. The extracted polarization *P*_r_ = 0.23 μC cm^–2^ is consistent with the theoretical predictions for sliding ferroelectric 3R-MoS_2_ ^10,32^ but one to two orders of magnitude smaller than those of other ferroelectric oxides.

The coexistence of weak polarization and giant TER motivates a broader comparison with reported FTJs. We therefore benchmark TER as a function of dipole switching energy density in oxide, intralayer vdW, and sliding ferroelectric FTJs (Figure 2d). The dipole switching energy density is estimated using *E*_sw_ = 2*P*_r_ *E*_c_ *d*, where *E*_c_ is the coercive field.^33^ For Au/bilayer 3R-MoS_2_/BDD FTJ, we obtain an ultralow dipole switching energy density of 4.31 fJ μm^–2^, using *P*_r_ = 0.23 μC cm^–2^, coercive field *E*_c_ = 0.67 V/nm extracted from CAFM measurements, and effective ferroelectric thickness *d* = 1.4 nm. Compared with prior FTJ platforms,^34–42^ the present sliding FTJ combines an ultralow switching energy density with an ultrahigh TER, deviating from the common trend (highlighted by the orange arrow) in which a larger TER is associated with a higher switching energy density. It points to a distinct mechanism beyond the simple polarization-controlled barrier height modulation.

The giant TER observed in Figure 2 is unexpected given the small polarization dipole and surface potential contrast of bilayer 3R-MoS_2_. We therefore asked whether polarization-induced barrier height modulation alone can account for the observed resistance contrast. Control junctions with the same bilayer 3R-MoS_2_ ferroelectric barrier but different contacts provide a direct test. KPFM measurements show comparable polarization-induced surface potential contrast in Pt and few-layer graphene control junctions (Supplementary Note 3), suggesting similar direct barrier height modulation. However, Au/3R-MoS_2_/Pt and Au/3R-MoS_2_/few-layer graphene FTJs exhibit TER values below 10, more than 4 orders of magnitude smaller than those of the Au/3R-MoS_2_/BDD FTJs (Figure 3a). These results rule out barrier height modulation alone as the origin of the giant TER, pointing to a more complex mechanism.

**Figure 3:**
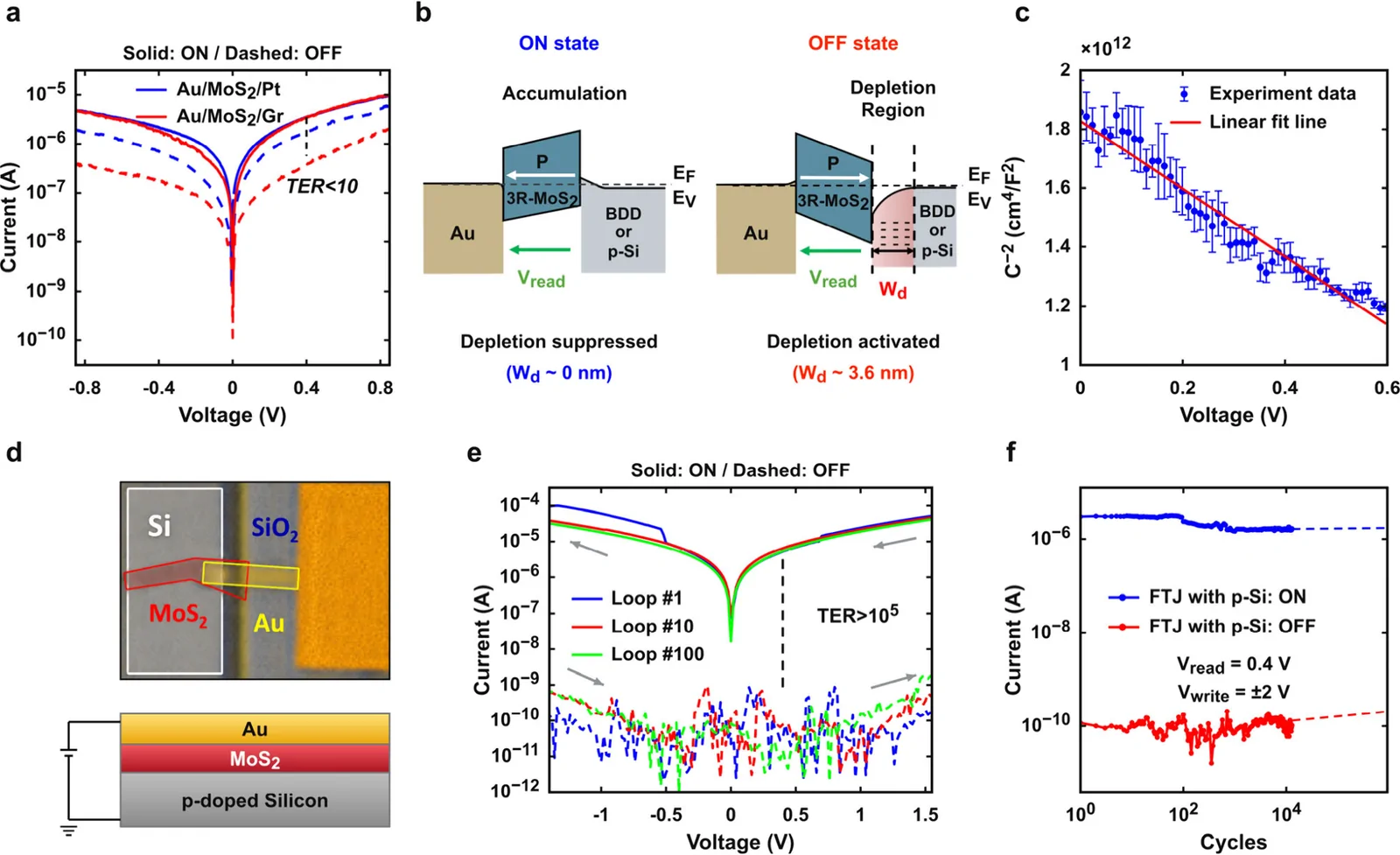
Interfacial depletion width modulation amplifies TER in sliding FTJs. **a**, Control *I*–*V* characteristics of the Au/3R-MoS_2_/Pt and Au/3R-MoS_2_/few-layer graphene FTJs. Both control devices show a TER less than 10, more than 4 orders of magnitude smaller than those in Au/3R-MoS_2_/BDD FTJs, indicating the barrier height modulation alone is insufficient to explain the giant TER. **b**, Schematic band diagrams of the Au/3R-MoS_2_/BDD or Au/3R-MoS_2_/p-Si FTJs in the ON and OFF states. Polarization switching modulates both the barrier height and the effective tunneling width through a polarization-dependent interfacial depletion region. In the ON state, the depletion region is suppressed, with *w*_d_ ∼ 0 nm; in the OFF state, the depletion region is activated, with *w*_d_ ∼ 3.6 nm. White arrows indicate the polarization directions, and the green arrows denote the direction of reading bias *V*_read_. **c**, *C* ^–2^–*V* characteristics of the Au/3R-MoS_2_/BDD FTJ in the OFF state. The linear behavior, shown by the red fit, is consistent with the depletion capacitance behavior and enables extraction of the interfacial depletion width. Error bars show the variance from 6 sets of data from repetitive SCM experiments. **d**, Planar device validation of the depletion-controlled sliding FTJ mechanism. Optical image of the fabricated device, with the bilayer 3R-MoS_2_ marked in red, Au electrode marked in yellow, and p-Si electrode marked with a white line. The schematic cross-section illustrates the vertical stacking of the Au/bilayer 3R-MoS_2_/p-Si junction used for two-terminal operation. **e**, Representative *I*–*V* characteristics of the Au/3R-MoS_2_/p-Si FTJ measured during repeated voltage sweeps. The first, 10th, and 100th loops are shown, with solid and dashed curves denoting the ON and OFF states, respectively. The overlapping loops show reproducible hysteretic tunneling with TER exceeding 10^5^. **f**, Endurance measurement over 10,000 switching cycles for the Au/3R-MoS_2_/p-Si FTJ while maintaining high TER. Extrapolation of the experimental trend suggests sustained high-contrast switching beyond 1 million cycles. Measurements were performed with *V*_read_ = 0.4 V and *V*_write_ = ±2 V.

To understand the additional amplification mechanism of TER, we consider tunneling through the Brinkman–Dynes–Rowell (BDR) model.^45^ The tunneling current density depends exponentially on both the average barrier height *ϕ̅* and the effective tunneling width *d* (Supplementary Note 4)­

1 $$J\propto \exp\left[-\frac{2\sqrt{2m\mathrm{\phi ̅}}}{\hbar }d\right]$$

where *m* is the effective carrier mass and ℏ is the reduced Planck constant. Even a nanoscale modulation of the effective tunneling width can strongly amplify the resistance contrast. Tunneling width variation can produce order-of-magnitude changes in tunneling current. This sensitivity suggests that sliding ferroelectrics may influence TER not only through electrostatic barrier modulation but also via changes in the effective tunneling width.

We tested this possibility using scanning capacitance microscopy (SCM) measurements on the Au/3R-MoS_2_/BDD junctions. The OFF state exhibits a linear dependence of capacitance *C*_d_^–2^ on applied bias *V*_d_ (Figure 3c), characteristic of depletion-type behavior at the interface between MoS_2_ and BDD. It is fitted by the following equation:^46^

2 $$C_{d}^{-2}=\frac{2\left(V_{\mathrm{bi}}-V_{d}\right)}{q\varepsilon_{0}\varepsilon_{r}N}$$

where *ε*_0_ represents the vacuum permittivity and *ε*_r_ denotes the relative permittivity of the BDD. Analysis yields a built-in potential *V*_bi_ = 1.83 V and carrier concentration *N* = 5 × 10^19^ cm^–3^, from which we extract a depletion width *w*_d_ = 3.6 nm at the interface of MoS_2_ and BDD using the formula^46^

3 $$w_{d}=\sqrt{\frac{2\varepsilon_{0}\varepsilon_{r}\left(V_{\mathrm{bi}}-V_{d}\right)}{qN}}$$

In contrast, the depletion region is strongly suppressed in the ON state without a linear dependence of capacitance *C*_d_^–2^ on applied bias *V*_d_. These results indicate that polarization reversal switches the interfacial depletion layer on and off, adding a polarization-dependent width contribution to the tunneling barrier. The mechanism is summarized schematically in Figure 3b. Sliding ferroelectric polarization modifies not only the ferroelectric barrier height but also the effective tunneling width. Combining the SCM-extracted width modulation with barrier height modulation measured from KPFM yields a calculated TER of ∼1 × 10^6^ (Supplementary Note 5), in good agreement with the experimental TER of 3.2 × 10^5^. This quantitative consistency confirms that the giant TER arises from the cooperative modulation of both barrier height and tunneling width, rather than from barrier height modulation alone. Our first-principles density functional theory (DFT) calculations also reveal the controllable depletion behavior at the p-Si side when switching from the AB state to the BA state (see Methods and Supplementary Note 7).

Having established the depletion width hybrid tunneling mechanism in the tip geometry of FTJs, we next tested whether using the same principle, planar sliding FTJs with high performance can be realized. For the planar FTJs, we employ the Au/Bilayer 3R-MoS_2_/p-Si heterostructures (Figure 3d), as SCM test results show that heavily doped p-Si (*N* = 2 × 10^19^ cm^–3^) can also support an interfacial depletion width comparable to that of BDD (Supplementary Note 6). The planar FTJ produces reproducible hysteretic tunneling with a TER exceeding 10^5^ (Figure 3e). Representative first, 10th, and 100th loops show nearly overlapping ON- and OFF-state curves, indicating stable switching without obvious sweep-to-sweep drift.

We also exclude the possibility that the hysteretic resistance switching could come from charge trapping, ion migration, or conductive filaments,^47–49^ where defect states, mobile ions, or local conductive paths can change the junction resistance under bias (see Supplementary Note 8). Those possible three mechanisms show large variance between cycles such as sweep-to-sweep drift, history-dependent current changes, or strong sweep rate dependence.^48,50^ It differs from the good repeatability found in the sliding FTJs, showing *I*–*V* loops overlapping very well (Figures 2a,b and 3e). The sweep-rate-dependent measurements show that TER has no observable dependence on sweep rate (Supplementary Figure S5a), meaning that there is a negligible contribution of charge trapping.

Furthermore, we tested the switching endurance of the planar sliding FTJs. The device remains stable over 10,000 switching cycles (Figure 3f). The TER remains above 10^4^ throughout the measured cycling tests, and extrapolation of the experimental trend suggests sustained high-contrast operation beyond 10^6^ switching events. Similar TER values were also obtained from multiple planar devices (Supplementary Figure S5b), indicating that the high-performance sliding FTJ is not a tip-specific effect and can be implemented in scalable and repeatable planar devices.

Although a giant TER is critical, scalable memory operation requires the resistance contrast to be spatially uniform across the junctions. We therefore investigate the microscopic origins of spatial variation in depletion-amplified sliding FTJs to help mitigate device variability. In bilayer 3R-MoS_2_, two sources of heterogeneity are particularly relevant: domain walls (DWs), which contribute to polarization reversal by mediating interlayer sliding,^14^ and residual strain, which is introduced during fabrication and can distort the potential landscape and modify polarization switching dynamics. To distinguish their roles, we compare two representative Au/3R-MoS_2_/BDD junctions: a nominally strain-free device containing domain walls, and a strained device in which local tunneling is measured within a single polarization domain while varying the local tensile strain. This comparison allows us to separate domain-wall-induced and strain-induced changes in the depletion-amplified tunneling contrast.

We first examine the role of domain walls in a strain-free device. PFM phase mapping confirms well-defined AB and BA domains written in the Au/3R-MoS_2_/BDD junction (see Methods), while Raman mapping of the *E*_2*g*_^1^ mode shows a negligible peak shift across the bilayer region (Figure 4a). The Raman peak remains close to the strain-free reference value of ∼384 cm^–1^, with spatial variations below 0.5 cm^–1^, indicating negligible strain below 0.2%.^51^ This allows us to isolate the effect of domain walls on the local tunneling behavior. Local *I*–*V* measurements acquired at different distances from the domain wall show reproducible hysteretic switching and large TER throughout the mapped region (Figure 4b). Quantitatively, TER decreases by approximately 1 order of magnitude with increasing distance from the domain wall but remains above 10^4^ even in the domain interior (Figure 4e).

**Figure 4:**
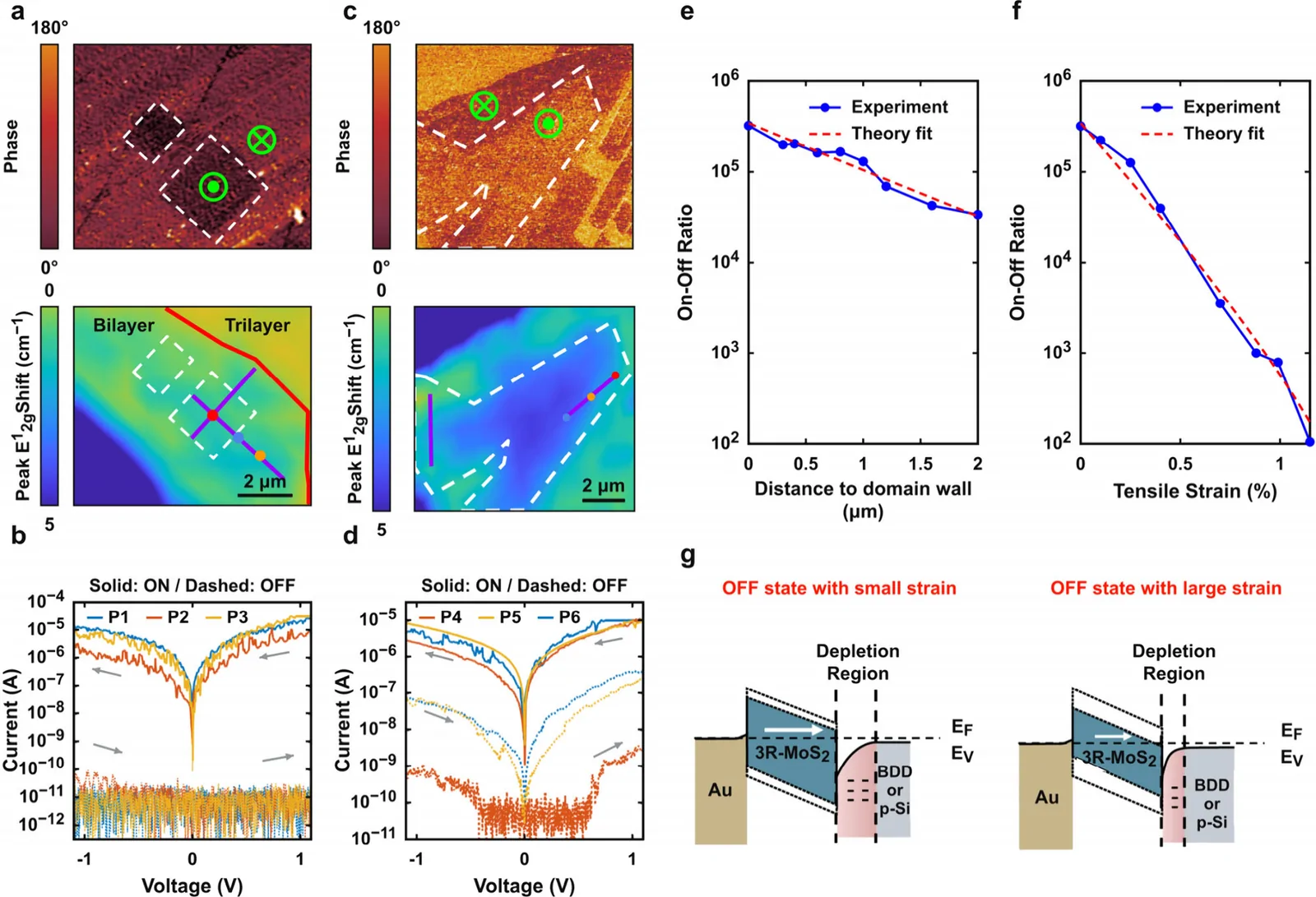
Strain governs the spatial uniformity of depletion-controlled TER. **a**,**c**, Spatial mapping of the strain-free (**a**) and strained (**c**) Au/3R-MoS_2_/BDD FTJs. Top: PFM phase maps showing ferroelectric domains with opposite out-of-plane polarizations with 180° phase contrast. The white dashed lines indicate boundaries between domains, and ⊙ and ⊗ denote polarization directions in AB and BA stackings, respectively. Bottom: Raman maps of the *E*_2*g*_^1^ peak shift acquired in the same regions. In the strain-free device (**a**), the bilayer region shows a negligible Raman peak shift, indicating minimal strain. In the strained device (**c**), the dark-blue region shows a Raman peak shift of up to ∼5 cm^–1^, corresponding to approximately 1.2% strain. Scale bars: 2 μm. **b**,**d**, Representative hysteretic *I*–*V* curves measured at points in (**a**) and (**c**), respectively. Solid and dashed curves denote the ON and OFF states, respectively, and gray arrows indicate the voltage sweep direction. In the strain-free device (**b**), positions P1–P3 sample different distances from the domain wall. In the strained device (**c**), positions P4–P6 sample regions with increasing local strain (0.3%, 0.6%, and 1.2%, respectively). **e**,**f**, Spatially resolved TER extracted from local *I*–*V* measurements as a function of distance from the domain wall (**e**) and local tensile strain (**f**). In the strain-free device, TER decreases by approximately 1 order of magnitude with increasing distance from the domain wall but remains above 10^4^. In contrast, increasing tensile strain suppresses TER by several orders of magnitude, from above 10^5^ to approximately 10^2^. Red dashed lines show fits to the height–width tunneling model. **g**, Schematic OFF-state band diagram illustrating the effect of tensile strain on both the barrier height and the width of the depletion region. Large tensile strain lowers the effective tunneling barrier and reduces the interfacial depletion contribution, thereby increasing the OFF-state current significantly and suppressing TER.

This distance-dependent TER variation can be rationalized by domain-wall-mediated switching in sliding ferroelectrics. In 3R-MoS_2_, opposite polarization domains are separated by partial dislocation walls, and polarization reversal is mediated by domain wall motion.^14,37,52^ Near an existing domain wall, interlayer sliding is energetically favored, and switching can reach completion within a narrow voltage window. In contrast, domain interiors require larger fields and can undergo incomplete switching.^25,37^ SS-PFM measurements (Supplementary Note 9) support this picture: positions near the domain wall show a full 180° phase reversal, while domain centers show only a ∼155° phase change, indicating partial switching. This incomplete switching reduces the effective barrier modulation by ∼20 meV (Supplementary Note 10), and incorporating this reduction into the height–width tunneling model reproduces the one-order-of-magnitude TER decrease away from the domain wall. Thus, domain walls enhance local switching completeness and tunneling modulation with TER above 10^5^, while the device as a whole maintains a high TER exceeding 10^4^.

We then isolate the effect of tensile strain by measuring a single-domain region with varying local strain while keeping the distance from the domain wall approximately fixed. As shown in Figure 4c, PFM mapping identifies the region AB stacking within a dashed white line, while spatial Raman mapping reveals a local *E*_2*g*_^1^ mode shift up to 5 cm^–1^. Using the reported strain-induced shift rate of 4 cm^–1^ per 1% strain,^51^ the strain in this device is up to 1.2% (Supplementary Note 11). Representative *I*–*V* curves show that the ON-state current remains comparatively stable, whereas the OFF-state current increases by several orders of magnitude with increasing strain (Figure 4d). Consequently, the TER decreases from above 10^5^ in low-strain regions to approximately 10^2^ in highly strained regions (Figure 4f).

Strain-induced suppression of TER can be understood through the combined impact of strain on both tunneling barrier height and depletion width. Our DFT calculations (Supplementary Note 10), together with previous studies, have shown that tensile strain narrows the bandgap of bilayer 3R-MoS_2_ by 120 meV per 1% strain.^53–55^ In sliding FTJs, this bandgap reduction lowers the tunneling barrier height in both the ON and OFF states, thereby enhancing carrier transmission. In addition to barrier lowering, tensile strain may also weaken the out-of-plane ferroelectric polarization through a strain-induced counter-polarization component^44,56^ (Supplementary Note 10). The reduced polarization can decrease the OFF-state depletion width and weaken the polarization-controlled tunneling width modulation. As a result, strain simultaneously lowers the barrier height and reduces the depletion width contribution to the OFF state (Figure 4g), leading to a disproportionately strong increase in the OFF current and consequent suppression of the TER. This picture is consistent with the experimental observation that the OFF current increases by roughly 3 orders of magnitude (from 10^–11^ A to 10^–8^ A) when applying 1% strain.

Theoretical calculations (Supplementary Note 10) model both the domain-wall-dependent and strain-dependent TER trends as shown by the red dashed lines in Figure 4e,f, in good agreement with experimental observations. For the strain-free device, the theoretical estimate captures the moderate TER decrease away from the domain wall by incorporating incomplete switching and reduced barrier modulation. For the strained device, the theoretical estimate reproduces the several-orders-of-magnitude TER suppression by lowering the strain-induced barrier and weakening the OFF-state depletion contribution. Taken together, these results show that strain is the primary factor driving the spatially inhomogeneous TER in sliding FTJs. Minimizing strain, and ideally employing low-strain sliding ferroelectric layers, is therefore necessary for achieving stable high-performance FTJs and mitigating device variance. We further examined the strain in the Au/3R-MoS_2_/p-Si device shown in Figure 3e to confirm the role of strain in a planar device. Raman mapping confirms that the device is nearly strain-free (Supplementary Note 12) and supports the device with low strain giving an ultrahigh TER (>10^5^).

## Conclusion

We demonstrate high-performance sliding FTJs based on bilayer 3R-MoS_2_ in both nanoscale tip geometries and lithographically defined planar devices. The junctions display reproducible current–voltage hysteresis with a giant TER exceeding 10^5^, despite the intrinsically weak out-of-plane polarization of sliding ferroelectricity. The reproduction of high switching contrast in multiple planar Au/3R-MoS_2_/p-Si devices confirms that the large TER is not a tip-specific effect but arises from the sliding ferroelectric junction itself. This performance places the device among the highest TER values reported for FTJs while preserving the ultralow switching energy characteristic of sliding ferroelectrics, thereby overcoming the conventional trend in which large TER is typically associated with large polarization and high dipole switching energy densities.

The large TER can be understood as a consequence of depletion-amplified height–width tunneling. Interlayer sliding changes the local electrostatic landscape at the MoS_2_–contact interface, producing a tunneling barrier profile that evolves not only in height but also in effective width. In particular, the small sliding polarization acts as a nanoscale electrostatic trigger that switches an interfacial depletion contribution on and off at depletion-capable contacts. Because tunneling transmission depends exponentially on the effective barrier width, this polarization-controlled width modulation amplifies the resistance contrast by several orders of magnitude. The stable ON and OFF currents observed in cycling tests further indicate that this mechanism can preserve the endurance advantage of sliding ferroelectrics. Additional measurements on the sliding FTJ at various temperatures (Supplementary Note 13) show a very weak dependence, indicating that direct tunneling dominates in this bilayer 3R-MoS_2_ FTJ instead of thermally activated tunneling, which provides a wide temperature operation range.

Spatially resolved measurements reveal that the uniformity of the depletion-amplified TER is governed primarily by local strain. Domain walls enhance local switching completeness and produce moderate TER variation, but the device maintains a high TER in low-strain regions. By contrast, tensile strain strongly suppresses the TER by modifying the local barrier profile and weakening the OFF-state depletion contribution, which increases the OFF-state tunneling current. These results identify strain management as a critical design rule for scalable sliding FTJs. Low-strain ferroelectric layers, controlled transfer, and interface engineering should therefore help preserve spatially uniform, high-contrast switching in future devices.^57–59^ Overall, our findings establish sliding ferroelectrics as a viable platform for ultrathin, low-energy, high-contrast nonvolatile memory. More broadly, this work shows that weak polarization does not limit tunneling electroresistance when coupled to an interface that converts small electrostatic changes into an amplified tunneling width modulation. When exploring other candidates for sliding FTJs, the carrier type, doping density, and screening length of the depletion-capable contacts should be carefully selected to enable the controllable depletion width. The strain tolerance and acceptable strain window need to be evaluated for FTJs based on new sliding ferroelectric materials. Overall, this work expands the device opportunities of sliding ferroelectricity for next-generation neuromorphic and in-memory computing technologies.

## Methods

### Heterostructure Fabrication

The Au/3R-MoS_2_ heterostructures were fabricated via a dry transfer process. 3R-MoS_2_ crystals were obtained from HQ Graphene. First, prepatterned bottom electrodes (2 nm Ti/10 nm Au) were defined on SiO_2_/Si substrates using a Heidelberg DWL 66+ laser writer, followed by e-beam evaporation. Bilayer 3R-MoS_2_ flakes were mechanically exfoliated onto SiO_2_/Si chips using scotch tape. The thickness of the flakes was identified from optical contrast and further confirmed by contact-mode atomic force microscopy. The selected flakes were then picked up using a polycarbonate (PC)-covered polydimethylsiloxane (PDMS) stamp and transferred onto the bottom metal electrodes by heating the SiO_2_/Si chip to 180 °C and then slowly lifting the stamp. Subsequently, contact-mode AFM was employed to verify bubble-free regions and to remove PC residues from the sample surface using a cantilever with a high force constant.

### Device with p-Si Electrode Fabrication

For the Au/3R-MoS_2_/p-Si devices, heavily p-doped silicon was used as the bottom electrode. The SiO_2_/Si substrates were first patterned using laser writing (Heidelberg DWL 66+) and RIE to remove the dielectric layer and expose the conductive Si active regions. The ferroelectric barrier consists of bilayer 3R-MoS_2_ mechanically exfoliated from bulk crystals onto a SiO_2_/Si substrate. The layer thickness was identified by AFM, and the ferroelectric domain orientations were prescreened using electrostatic force microscopy (EFM). The device assembly was completed using a dry transfer method. First, the top Au electrodes (20 nm) were defined on a separate substrate. A PC/PDMS stamp was then used to pick up the Au electrodes, followed by picking up the target bilayer 3R-MoS_2_ flake. The entire stack (Au/3R-MoS_2_) was then transferred onto the exposed Si window of the substrate at 180 °C, resulting in a Si/3R-MoS_2_/Au vertical junction. Finally, the PC residue was removed using chloroform.

### Raman Measurement

The Raman spectra mappings were obtained using a Horiba/AIST-NT LabRAM system. A linearly polarized laser beam with a wavelength of 532 nm was directed and focused onto the samples using a 100× objective lens with a numerical aperture of 0.9. The same lens captured the scattered Raman signals and directed them back to the spectrometer. The step size of the Raman mapping was set to 0.5 μm.

### AFM, PFM, CAFM, and KPFM Measurements

The surface morphology of the FTJ heterostructures was characterized by AFM using an Asylum Research Cypher S AFM with AC160 tips in contact mode. KPFM was carried out to map the surface potential and determine the polarization directions. Single-frequency PFM mode was employed to acquire the spatial maps of phase and amplitude for ferroelectric domains. SS-PFM measurements were performed in the dual AC resonance tracking (DART) mode to measure the local switching behavior across different domains on the FTJ, with triangular pulse waveforms applied to the tip. CAFM measurements were also conducted on the Cypher S system using All-In-One-DD (AIO-DD) tips, with bias applied to the tip to record local tunneling *I*–*V* curves and measure the tunneling current under bias. SCM measurements were performed on a Bruker Dimension Icon system, using both AIO-DD and ElectriAll-In-One (AIOE) tips, to measure the capacitance behavior and extract the depletion width. Calibration was carried out with the Bruker SCM test sample.

### First-Principles Calculations

Strain-dependent bandgap was calculated using first-principles density functional theory^60,61^ as implemented in Vienna Ab Initio Simulation Package (VASP)^62^ with the projector augmented wave method,^63^ the Perdew–Burke–Ernzerhof (PBE)^64^ exchange-correlation energy functional, and Grimme’s DFT-D3 method^65^ with zero-damping function for van der Waals interaction. The large bilayer 3R-MoS_2_/p-Si interface was calculated using OpenMX^66–68^ with the PBE functional, which consisted of a 5 × 5 bilayer 3R-MoS_2_ supercell and an H-terminated Si (111) slab with a total of 342 atoms. More details can be found in Supplementary Notes 7 and 10.

### Domain Writing

We first employed vertical PFM to confirm the presence of two opposite out-of-plane polarization states, separated by well-defined domain walls. To induce ferroelectric switching, a vertical electric field was applied by adding a DC bias voltage of +5 V through the tip to the sample. This electric field triggered the lateral propagation of the domain walls, thereby switching the polarization. We wrote a 1.75 μm × 1.75 μm box with bias voltage on the domain and domain wall regions, which expanded the domain boundary and flipped the polarization. A subsequent larger-area PFM scan without DC bias was performed right after this to confirm the artificial ferroelectric domain. To minimize potential strain effects from the contact-mode operation, the domain writing scans were performed at a reduced set point.

## Supplementary Material



## Data Availability

The data that support the plots within this paper and other findings of this study are available from the corresponding author upon reasonable request.

## Notes

AFM and KPFM characterization of bilayer 3R-MoS_2_; surface potential characterization of Pt- and graphene-contacted control junctions; tunneling and depletion width analyses; SCM characterization of BDD- and p-Si-contacted FTJs; first-principles calculations of polarization-dependent charge redistribution at the MoS_2_/p-Si interface; sweep rate dependence and device-to-device reproducibility; SS-PFM characterization; theoretical and experimental analyses of strain- and domain-wall-dependent TER, including strain-dependent bandgap calculations and Raman/KPFM strain–polarization correlations; EFM and Raman characterization of planar FTJs; and temperature-dependent *I*–*V* measurements (PDF)

## References

[1] Xia Q., Yang J. J. (2019). Memristive
Crossbar Arrays for Brain-Inspired Computing. Nat. Mater..

[2] Kohlstedt H., Pertsev N. A., Rodríguez Contreras J., Waser R. (2005). Theoretical
Current-Voltage Characteristics of Ferroelectric Tunnel Junctions. Phys. Rev. B.

[3] Garcia V., Bibes M. (2014). Ferroelectric Tunnel Junctions for Information Storage and Processing. Nat. Commun..

[4] Tsymbal E. Y., Kohlstedt H. (2006). Tunneling
Across a Ferroelectric. Science.

[5] Chanthbouala A., Crassous A., Garcia V., Bouzehouane K., Fusil S., Moya X., Allibe J., Dlubak B., Grollier J., Xavier S., Deranlot C., Moshar A., Proksch R., Mathur N. D., Bibes M., Barthélémy A. (2012). Solid-State
Memories Based on Ferroelectric Tunnel Junctions. Nat. Nanotechnol..

[6] Scott J. F. (2007). Applications
of Modern Ferroelectrics. Science.

[7] Khan A. I., Keshavarzi A., Datta S. (2020). The Future of Ferroelectric Field-Effect
Transistor Technology. Nat. Electron..

[8] Schroeder U., Park M. H., Mikolajick T., Hwang C. S. (2022). The Fundamentals
and Applications of Ferroelectric HfO_2_. Nat. Rev. Mater..

[9] Fox C., Mao Y., Zhang X., Wang Y., Xiao J. (2024). Stacking Order
Engineering
of Two-Dimensional Materials and Device Applications. Chem. Rev..

[10] Li L., Wu M. (2017). Binary Compound Bilayer and Multilayer with Vertical
Polarizations:
Two-Dimensional Ferroelectrics, Multiferroics, and Nanogenerators. ACS Nano.

[11] Ji J., Yu G., Xu C., Xiang H. J. (2023). General Theory for Bilayer Stacking
Ferroelectricity. Phys. Rev. Lett..

[12] Wu M., Li J. (2021). Sliding Ferroelectricity
in 2D van der Waals Materials: Related Physics
and Future Opportunities. Proc. Natl. Acad.
Sci. U.S.A..

[13] Liang J., Yang D., Wu J., Xiao Y., Watanabe K., Taniguchi T., Dadap J. I., Ye Z. (2025). Resolving Polarization
Switching Pathways of Sliding Ferroelectricity in Trilayer 3R-MoS_2_. Nat. Nanotechnol..

[14] Yang D., Liang J., Wu J., Xiao Y., Dadap J. I., Watanabe K., Taniguchi T., Ye Z. (2024). Non-Volatile Electrical
Polarization Switching *via* Domain Wall Release in
3R-MoS_2_ Bilayer. Nat. Commun..

[15] Meng P., Wu Y., Bian R., Pan E., Dong B., Zhao X., Chen J., Wu L., Sun Y., Fu Q., Liu Q., Shi D., Zhang Q., Zhang Y.-W., Liu Z., Liu F. (2022). Sliding Induced Multiple
Polarization States in Two-Dimensional Ferroelectrics. Nat. Commun..

[16] Fei Z., Zhao W., Palomaki T. A., Sun B., Miller M. K., Zhao Z., Yan J., Xu X., Cobden D. H. (2018). Ferroelectric
Switching of a Two-Dimensional Metal. Nature.

[17] Xiao J., Wang Y., Wang H., Pemmaraju C. D., Wang S., Muscher P., Sie E. J., Nyby C. M., Devereaux T. P., Qian X., Zhang X., Lindenberg A. M. (2020). Berry Curvature
Memory through Electrically Driven Stacking Transitions. Nat. Phys..

[18] Yasuda K., Wang X., Watanabe K., Taniguchi T., Jarillo-Herrero P. (2021). Stacking-Engineered Ferroelectricity in Bilayer Boron
Nitride. Science.

[19] Vizner
Stern M., Waschitz Y., Cao W., Nevo I., Watanabe K., Taniguchi T., Sela E., Urbakh M., Hod O., Ben Shalom M. (2021). Interfacial Ferroelectricity by van der Waals Sliding. Science.

[20] Wang X., Yasuda K., Zhang Y., Liu S., Watanabe K., Taniguchi T., Hone J., Fu L., Jarillo-Herrero P. (2022). Interfacial
Ferroelectricity in Rhombohedral-Stacked Bilayer Transition Metal
Dichalcogenides. Nat. Nanotechnol..

[21] Yasuda K., Zalys-Geller E., Wang X., Bennett D., Cheema S. S., Watanabe K., Taniguchi T., Kaxiras E., Jarillo-Herrero P., Ashoori R. (2024). Ultrafast High-Endurance Memory Based on Sliding Ferroelectrics. Science.

[22] Ma C., Luo Z., Huang W., Zhao L., Chen Q., Lin Y., Liu X., Chen Z., Liu C., Sun H., Jin X., Yin Y., Li X. (2020). Sub-Nanosecond Memristor Based on Ferroelectric Tunnel
Junction. Nat. Commun..

[23] Bian R., He R., Pan E., Li Z., Cao G., Meng P., Chen J., Liu Q., Zhong Z., Li W., Liu F. (2024). Developing Fatigue-Resistant Ferroelectrics Using Interlayer
Sliding
Switching. Science.

[24] Choi K. J., Biegalski M., Li Y. L., Sharan A., Schubert J., Uecker R., Reiche P., Chen Y. B., Pan X. Q., Gopalan V., Chen L.-Q., Schlom D. G., Eom C. B. (2004). Enhancement
of Ferroelectricity in Strained BaTiO_3_ Thin Films. Science.

[25] Lv M., Sun X., Chen Y., Taniguchi T., Watanabe K., Wu M., Wang J., Xue J. (2022). Spatially Resolved Polarization Manipulation
of Ferroelectricity in Twisted hBN. Adv. Mater..

[26] Lv M., Wang J., Tian M., Wan N., Tong W., Duan C., Xue J. (2024). Multiresistance States
in Ferro-
And Antiferroelectric Trilayer Boron Nitride. Nat. Commun..

[27] Strachan J., Masters A. F., Maschmeyer T. (2021). 3R-MoS_2_ in Review: History,
Status, and Outlook. ACS Appl. Energy Mater..

[28] Jiang H., Li L., Wu Y., Duan R., Yi K., Wu L., Zhu C., Luo L., Xu M., Zheng L., Gan X., Zhao W., Wang X., Liu Z. (2024). Vapor Deposition of
Bilayer 3R MoS_2_ with Room-Temperature Ferroelectricity. Adv. Mater..

[29] Wan S., Huang H., Liu H., Liu H., Li Z., Li Y., Liao Z., Lanza M., Zeng H., Zhou Y. (2024). Intertwined
Flexoelectricity and Stacking Ferroelectricity in Marginally Twisted
hBN Moiré Superlattice. Adv. Mater..

[30] Cui C., Xue F., Hu W.-J., Li L.-J. (2018). Two-Dimensional Materials with Piezoelectric
and Ferroelectric Functionalities. npj 2D Mater.
Appl..

[31] Deb S., Cao W., Raab N., Watanabe K., Taniguchi T., Goldstein M., Kronik L., Urbakh M., Hod O., Ben Shalom M. (2022). Cumulative
Polarization in Conductive Interfacial Ferroelectrics. Nature.

[32] Park J., Yeu I. W., Han G., Hwang C. S., Choi J.-H. (2019). Ferroelectric
Switching in Bilayer 3R MoS_2_
*via* Interlayer
Shear Mode Driven by Nonlinear Phononics. Sci.
Rep..

[33] Paruch P., Giamarchi T., Tybell T., Triscone J.-M. (2006). Nanoscale Studies
of Domain Wall Motion in Epitaxial Ferroelectric Thin Films. J. Appl. Phys..

[34] Pantel D., Lu H., Goetze S., Werner P., Kim D. J., Gruverman A., Hesse D., Alexe M. (2012). Tunnel Electroresistance in Junctions
with Ultrathin Ferroelectric Pb­(Zr_0.2_Ti_0.8_)­O_3_ Barriers. Appl. Phys. Lett..

[35] Lu H., Lipatov A., Ryu S., Kim D. J., Lee H., Zhuravlev M. Y., Eom C. B., Tsymbal E. Y., Sinitskii A., Gruverman A. (2014). Ferroelectric Tunnel Junctions with Graphene Electrodes. Nat. Commun..

[36] Yamada H., Garcia V., Fusil S., Boyn S., Marinova M., Gloter A., Xavier S., Grollier J., Jacquet E., Carrétéro C., Deranlot C., Bibes M., Barthélémy A. (2013). Giant Electroresistance
of Super-Tetragonal
BiFeO_3_-Based Ferroelectric Tunnel Junctions. ACS Nano.

[37] Gao Y., Weston A., Enaldiev V., Li X., Wang W., Nunn J. E., Soltero I., Castanon E. G., Carl A., De Latour H., Summerfield A., Hamer M., Howarth J., Clark N., Wilson N. R., Kretinin A. V., Fal’ko V. I., Gorbachev R. (2024). Tunnel Junctions Based on Interfacial Two Dimensional
Ferroelectrics. Nat. Commun..

[38] Yuan S., Luo X., Chan H. L., Xiao C., Dai Y., Xie M., Hao J. (2019). Room-Temperature Ferroelectricity
in MoTe_2_ Down to the
Atomic Monolayer Limit. Nat. Commun..

[39] Wu J., Chen H.-Y., Yang N., Cao J., Yan X., Liu F., Sun Q., Ling X., Guo J., Wang H. (2020). High Tunnelling
Electroresistance in a Ferroelectric van der Waals Heterojunction *via* Giant Barrier Height Modulation. Nat. Electron..

[40] Luo Y., Chen J., Abbas A., Li W., Sun Y., Sun Y., Yi J., Lin X., Qiu G., Wen R., Chai Y., Liang Q., Zhou C. (2024). Robust Giant Tunnel
Electroresistance and Negative Differential Resistance in 2D Semiconductor/α-In_2_Se_3_ Ferroelectric Tunnel Junctions. Adv. Funct. Mater..

[41] Jia Y., Yang Q., Fang Y.-W., Lu Y., Xie M., Wei J., Tian J., Zhang L., Yang R. (2024). Giant Tunnelling Electroresistance
in Atomic-Scale Ferroelectric Tunnel Junctions. Nat. Commun..

[42] Luo Q., Cheng Y., Yang J., Cao R., Ma H., Yang Y., Huang R., Wei W., Zheng Y., Gong T., Yu J., Xu X., Yuan P., Li X., Tai L., Yu H., Shang D., Liu Q., Yu B., Ren Q. (2020). A Highly CMOS Compatible Hafnia-Based Ferroelectric
Diode. Nat. Commun..

[43] Lv X., Taniguchi T., Watanabe K., Lv M., Xue J. (2025). Electronic
Density of States Modulated Sliding Ferroelectric Tunneling Junctions. Phys. Rev. B.

[44] Mu W., Ke C., Guo N., Zhang Z., Yang L., Li Z., Zhou Y., Li J., Yue S., Zheng J., Zheng Q., Liu S., Jiao L. (2026). Sliding Ferroelectricity
in MoS_2_/ReS_2_ Heterojunction for a Sensitive
and Reconfigurable Bioelectronic Interface. ACS Nano.

[45] Gruverman A., Wu D., Lu H., Wang Y., Jang H. W., Folkman C. M., Zhuravlev M. Ye., Felker D., Rzchowski M., Eom C.-B., Tsymbal E. Y. (2009). Tunneling Electroresistance Effect
in Ferroelectric Tunnel Junctions at the Nanoscale. Nano Lett..

[46] Xi Z., Ruan J., Li C., Zheng C., Wen Z., Dai J., Li A., Wu D. (2017). Giant Tunnelling Electroresistance
in Metal/Ferroelectric/Semiconductor Tunnel Junctions by Engineering
the Schottky Barrier. Nat. Commun..

[47] Waser R., Aono M. (2007). Nanoionics-Based Resistive
Switching Memories. Nat. Mater..

[48] Sun W., Gao B., Chi M., Xia Q., Yang J. J., Qian H., Wu H. (2019). Understanding Memristive
Switching *via in situ* Characterization
and Device Modeling. Nat. Commun..

[49] Yang Y., Gao P., Gaba S., Chang T., Pan X., Lu W. (2012). Observation
of Conducting Filament Growth in Nanoscale Resistive Memories. Nat. Commun..

[50] Yang Y., Wu M., Zheng X., Zheng C., Xu J., Xu Z., Li X., Lou X., Wu D., Liu X., Pennycook S. J., Wen Z. (2021). Atomic-Scale Fatigue Mechanism of Ferroelectric Tunnel Junctions. Sci. Adv..

[51] Lee J.-U., Woo S., Park J., Park H. C., Son Y.-W., Cheong H. (2017). Strain-Shear
Coupling in Bilayer MoS_2_. Nat. Commun..

[52] Liang J., Yang D., Xiao Y., Chen S., Dadap J. I., Rottler J., Ye Z. (2023). Shear Strain-Induced
Two-Dimensional
Slip Avalanches in Rhombohedral MoS_2_. Nano Lett..

[53] Conley H. J., Wang B., Ziegler J. I., Haglund R. F., Pantelides S. T., Bolotin K. I. (2013). Bandgap Engineering
of Strained Monolayer and Bilayer MoS_2_. Nano Lett..

[54] Manzeli S., Allain A., Ghadimi A., Kis A. (2015). Piezoresistivity and
Strain-Induced Band Gap Tuning in Atomically Thin MoS_2_. Nano Lett..

[55] Bhattacharyya S., Pandey T., Singh A. K. (2014). Effect of Strain
on Electronic and
Thermoelectric Properties of Few Layers to Bulk MoS_2_. Nanotechnology.

[56] Duerloo K.-A. N., Ong M. T., Reed E. J. (2012). Intrinsic Piezoelectricity in Two-Dimensional
Materials. J. Phys. Chem. Lett..

[57] Hong S. C., Baek J. H., Kim J., Lee H., Lee G. H. (2025). Strain-Mediated
Control of Domain Structures in 3R-MoS_2_ Bilayers Synthesized
by Vapor-Liquid-Solid Growth in Chemical Vapor Deposition. ACS Nano.

[58] Chen T.-A., Chuu C.-P., Tseng C.-C., Wen C.-K., Wong H.-S. P., Pan S., Li R., Chao T.-A., Chueh W.-C., Zhang Y., Fu Q., Yakobson B. I., Chang W.-H., Li L.-J. (2020). Wafer-Scale Single-Crystal Hexagonal Boron Nitride Monolayers on
Cu (111). Nature.

[59] Kang K., Xie S., Huang L., Han Y., Huang P. Y., Mak K. F., Kim C. J., Muller D., Park J. (2015). High-Mobility Three-Atom-Thick
Semiconducting Films with Wafer-Scale Homogeneity. Nature.

[60] Hohenberg P., Kohn W. (1964). Inhomogeneous Electron Gas. Phys. Rev..

[61] Kohn W., Sham L. J. (1965). Self-Consistent
Equations Including Exchange and Correlation
Effects. Phys. Rev..

[62] Kresse G., Furthmüller J. (1996). Efficient
Iterative Schemes for *ab initio* Total-Energy Calculations
Using a Plane-Wave Basis Set. Phys. Rev. B.

[63] Blöchl P. E. (1994). Projector
Augmented-Wave Method. Phys. Rev. B.

[64] Perdew J. P., Burke K., Ernzerhof M. (1996). Generalized
Gradient Approximation
Made Simple. Phys. Rev. Lett..

[65] Grimme S., Antony J., Ehrlich S., Krieg H. (2010). A Consistent and Accurate *ab initio* Parametrization
of Density Functional Dispersion
Correction (DFT-D) for the 94 Elements H-Pu. J. Chem. Phys..

[66] Ozaki T., Kino H. (2004). Numerical Atomic Basis
Orbitals from H to Kr. Phys. Rev. B.

[67] Ozaki T., Kino H. (2005). Efficient Projector
Expansion for the *ab initio* LCAO
Method. Phys. Rev. B.

[68] Ozaki T. (2003). Variationally
Optimized Atomic Orbitals for Large-Scale Electronic Structures. Phys. Rev. B.

