# Supplementary material for: Sliding Ferroelectric Tunnel Junctions with Ultrahigh Tunneling Electroresistance
Source: ACS Nano. 2026 Oct 20;20(43):29004–14. doi: 10.1021/acsnano.6c11972 (PMC13738781; doi:10.1021/acsnano.6c11972)
Supplement: Supplementary file 1 [file nn6c11972_si_001.pdf]

Supporting Information: Sliding Ferroelectric Tunnel Junctions with Ultrahigh Tunneling Electroresistance

Haolin You[1†], Chen Shao[1†], Saagar Kolachina[2], Adrian Jonathan Lim[1], Robert Boyd[3,5], Chengdong Wang[2], Fan Fei[3], Carter Fox[4], Sina Najmaei[5], Jun Xiao[1,3,4], Daniel A. Rhodes[3,4], Xiaofeng Qian[2,6,7], Ying Wang[1,3,4*]

[1]Department of Electrical and Computer Engineering, University of Wisconsin–Madison, 1415 Engineering Dr., Madison, 53706, WI, USA.

[2]Department of Materials Science and Engineering, Texas A&M University, 575 Ross St., College Station, 77843, TX, USA.

[3]Department of Materials Science and Engineering, University of Wisconsin–Madison, 1509 University Ave., Madison, 53706, WI, USA.

[4]Department of Physics, University of Wisconsin–Madison, 1150 University Ave., Madison, 53706, WI, USA.

[5]DEVCOM Army Research Laboratory, Adelphi Laboratory Center, Adelphi, Maryland, 20783, USA.

[6]Department of Physics and Astronomy, Texas A&M University, 578 University Dr., College Station, 77843, TX, USA.

[7]Department of Electrical and Computer Engineering, Texas A&M University, 188 Bizzell St., College Station, 77843, TX, USA.

*Corresponding author E-mail: y.wang@wisc.edu;

[†]These authors contributed equally to this work.

Supplementary Note 1: AFM Characterization of Bilayer 3R-MoS_2

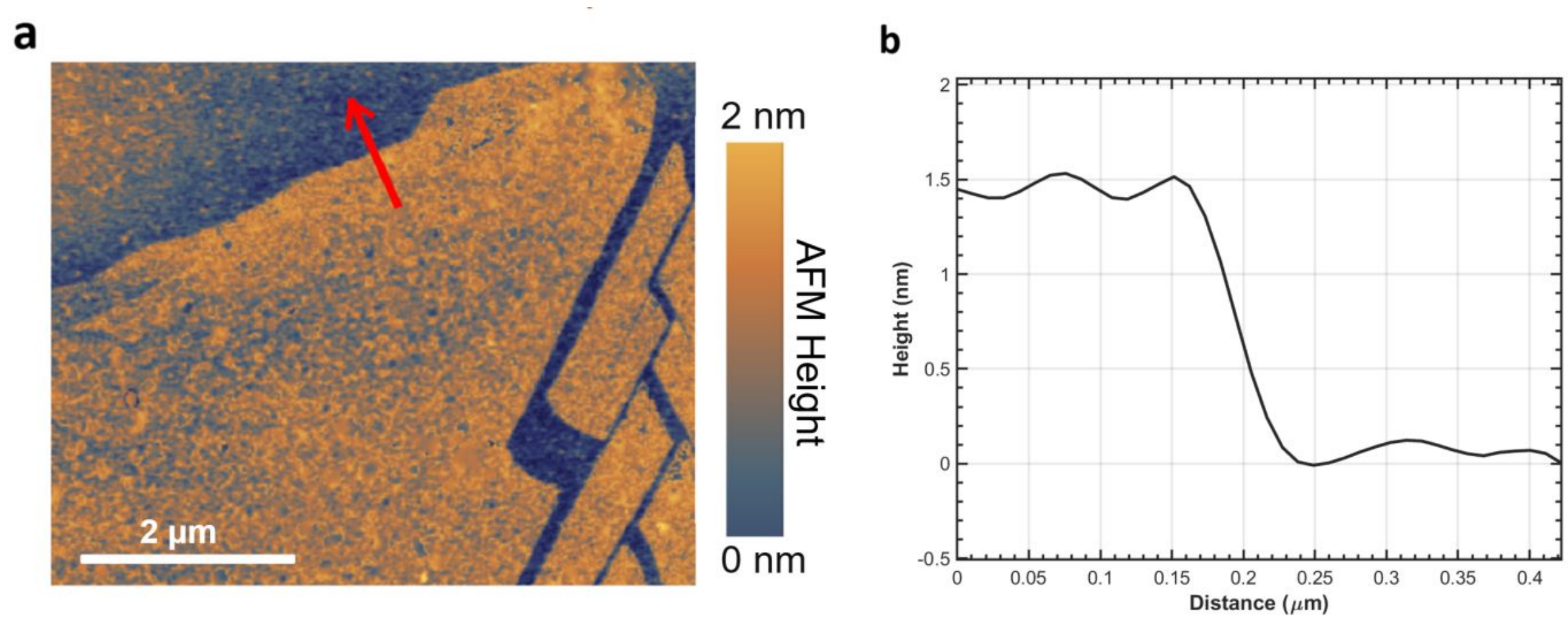

Fig. S1: AFM characterization of bilayer 3R-MoS_2. (a) AFM height mapping image of a bilayer 3R-MoS_2 on top of gold contact. (b) Corresponding line-cut profile taken along the red line in (a), showing a step height of ~ 1.4 nm, confirming the MoS_2 flake is bilayer.

We first performed AFM (using contact mode in Asylum Research Cypher S AFM Microscope) to verify the thickness and quality of the MoS_2 flakes used in our devices. Fig. S1 shows a typical AFM mapping and line profile set of a MoS_2 flake on top of gold contact. The thickness of the bilayer MoS_2 is about 1.4 nm, read from the line profile.

Supplementary Note 2: KPFM Confirmation of Polarization

Kelvin Probe Force Microscopy (KPFM) measurement was employed to resolve the surface-potential contrast between different ferroelectric domains. The relationship between

the measured contact potential difference (V_{CPD}) and the sample's work function (ϕ_{sample}) is governed by the equation:

$$V_{\mathrm{CPD}} = \frac{\phi_{\mathrm{tip}} - \phi_{\mathrm{sample}}}{-e} \text{(S1)}$$

According to this relation, a higher V_{CPD} (bright area in Fig. 2c) corresponds to a higher work function stacking of the sample, while a lower V_{CPD} (dark area in Fig. 2c) indicates a lower work function stacking of the sample. Then we can link these work function variations to the ferroelectric polarization direction based on previous KPFM study. [1] Specifically, upward polarization (P↑) accumulates positive bound charges at the MoS_2 surface and leads to lower work function. Consequently, we assign the darker domains (lower ϕ_{sample}) to the P↑ state. Conversely, downward polarization (P↓) induces negative bound charges at the surface, leading to a higher work function, appearing as brighter domains in the KPFM phase image. The polarization direction of other MoS_2 flakes is also confirmed using this established method.

Supplementary Note 3: Surface Potential Characterization of Devices with Pt and Graphene Electrodes

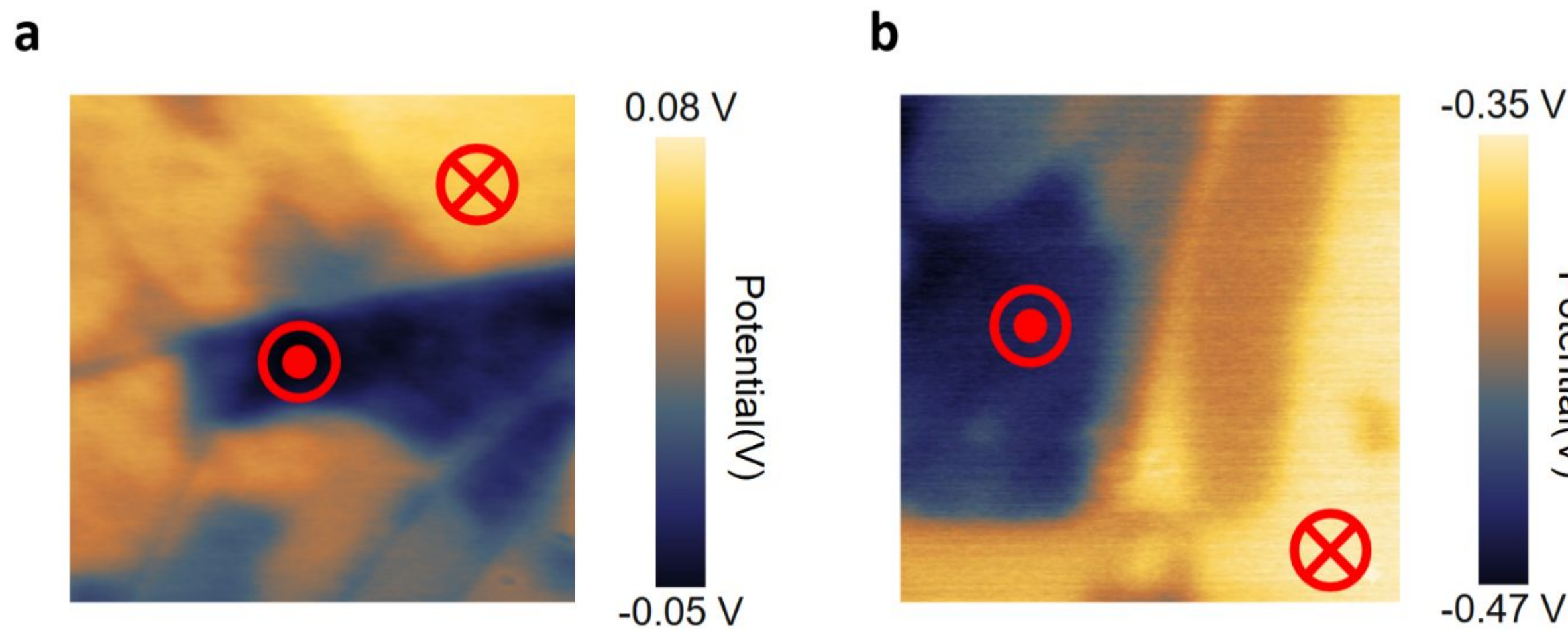

Fig. S2: KPFM characterization of sliding FTJs with Pt and Graphene electrodes. (a-b), KPFM surface potential maps of AB/BA (marked by ⊙ and ⊗, respectively) in the Au/3R-MoS_2/Pt (a) and Au/3R-MoS_2/Graphene (b) heterostructures. Both control devices exhibit a polarization-dependent surface potential contrast comparable to that of the BDD-contacted FTJ (130 mV).

To verify that the giant TER in our sliding FTJs does not solely originate from polarization-induced barrier height modulation, we performed KPFM on control devices utilizing Pt and few-layer graphene (Gr) top electrodes, as shown in Fig. S2. The calculated barrier height modulation differences of Au/3R-MoS_2/Pt and Au/3R-MoS_2/Gr FTJs are 0.121 eV and 0.125 eV, indicating a similar interfacial barrier height modulation of 0.130 eV measured from BDD tip. However, despite this comparable barrier height modulation, the TER for devices with Pt or Graphene electrode remains low, which is below 10 (Fig. 3a). This striking performance contrast suggests that barrier height modulation alone is insufficient to produce the giant TER.

Supplementary Note 4: Tunneling Model and Depletion Analysis

Tunneling transport through an asymmetric trapezoidal barrier of thickness d, with barrier heights on interfaces ϕ_1 (Au interface) and ϕ_2 (BDD interface), is modeled using the Brinkman–Dynes–Rowell (BDR) framework:[2]

$$J \approx C \frac{\exp\left\{\alpha(V)\left[\left(\phi_2 - \frac{eV}{2}\right)^{\frac{3}{2}} - \left(\phi_1 + \frac{eV}{2}\right)^{\frac{3}{2}}\right]\right\}}{\alpha(V)^2\left[\left(\phi_2 - \frac{eV}{2}\right)^{\frac{1}{2}} - \left(\phi_1 + \frac{eV}{2}\right)^{\frac{1}{2}}\right]^2} \times \sinh\left\{\frac{3}{2}\alpha(V)\left[\left(\phi_2 - \frac{eV}{2}\right)^{\frac{1}{2}} - \left(\phi_1 + \frac{eV}{2}\right)^{\frac{1}{2}}\right]\frac{eV}{2}\right\}, \quad \text{(S2)}$$

where $\alpha(V) = 4d\sqrt{2m}/3\hbar eV$ and $C = -\,4em/9\pi^2\hbar^3$.

The tunneling current is exponentially sensitive to the effective barrier height ϕ and width w_d. Accordingly, a simplified exponential form is sufficient to describe polarization-induced current modulation and tunneling electroresistance.

In the nearly symmetric barrier limit ($|\phi_2 - \phi_1| < \overline{\phi}$), a first-order expansion in V yields:[2]

$$J \approx \frac{e^2}{h}\,\frac{1}{2\pi d}\,\frac{\sqrt{2m\overline{\phi}}}{\hbar}\,\exp\left[-\frac{2\sqrt{2m\overline{\phi}}}{\hbar}d\right] \quad \text{(S3)}$$

which simplifies to the exponential dependence given in Eq. (1) of the main text. The tunneling current density is more sensitive to the exponential terms and can be approximated as

$$J \propto \exp\left[-\frac{2\sqrt{2m\bar{\phi}}}{\hbar}d\right],$$

demonstrating that the combined modulation of the barrier height $\bar{\phi}$ and effective width d can exponentially amplify the tunneling current contrast.

Supplementary Note 5: Correlation between SCM-derived Depletion Width and CAFM-measured TER

To verify that the "height–width" hybrid model (polarization modified not only the barrier height but also the depletion width) is the origin of the large TER in the Au/3R-MoS_2/BDD FTJs, we use the depletion-width modulation extracted from SCM measurements to calculate the theoretical TER and compare it with the TER we measured from CAFM. SCM measurements were conducted using a Bruker Icon system under ambient conditions. The local C_{d}^{-2}–V_{d} plot (Fig. 3c) of the OFF state exhibits a clear linear relationship, consistent with depletion behavior.[3] From the linear fit, the built-in potential V_{bi} was determined to be approximately 1.83 V. According to the depletion approximation,

$$w_{\mathrm{d}} = \sqrt{\frac{2\varepsilon_{\mathrm{s}}(V_{\mathrm{bi}} - V_{\mathrm{d}})}{qN}},$$

the depletion width at the read voltage ($V_{\mathrm{d}} = 1$ V) was estimated to be $w_{\mathrm{d,off}} \approx 3.6$ nm, assuming $\varepsilon_{\mathrm{s}} = 6\,\varepsilon_0$ and $N = 5 \times 10^{19}\ \mathrm{cm}^{-3}$ on the BDD interface. In contrast, the depletion region in the ON state nearly disappears ($w_{\mathrm{d,on}} \approx 0$), consistent with the emergence of a strong tunneling channel and a low-resistance state. To verify the

consistency between the theoretical TER calculated from the SCM-derived parameters and the measured TER, we employed the tunneling current model mentioned in the main content:

$$J \propto \exp[-K\sqrt{\phi}\, d_{\mathrm{eff}}], \quad K = \frac{2\sqrt{2m^*}}{\hbar},$$

where $d_{\mathrm{eff}} = d_{\mathrm{FE}} + w_{\mathrm{d}}$ is the total effective barrier thickness and ϕ is the barrier height. Using $m^* = 0.5\, m_0$, $\phi_{\mathrm{on}} = 0.25$ eV, $\phi_{\mathrm{off}} = 0.37$ eV (Based on KPFM measurements), $d_{\mathrm{FE}} = 1.4$ nm (thickness of bilayer 3R-MoS_2), and $w_{\mathrm{d,off}} = 3.6$ nm (Calculated with the above SCM measurements), the calculated tunneling current ratio

$$\mathrm{TER} = \frac{J_{\mathrm{on}}}{J_{\mathrm{off}}} = \exp\left[K\left(\sqrt{\phi_{\mathrm{off}}}\, d_{\mathrm{off}} - \sqrt{\phi_{\mathrm{on}}}\, d_{\mathrm{on}}\right)\right] \text{(S4)}$$

yields a value of 1.0×10^6, which shows the potential of the FTJ could be better than the experimentally measured TER of 3.2×10^5. Notably, the experimentally measured TER is limited by the detection floor of our CAFM setup: the OFF-state current is bounded by the instrument noise floor at $\sim 10^{-10}$ A (tunneling currents below this value are not resolved). As a result, the measured TER saturates at $\sim 3.2 \times 10^5$, whereas SCM results and estimation indicate that the OFF current can be lower and the intrinsic TER might have space to improve. This quantitative agreement confirms that the observed TER originates from the cooperative modulation of both the barrier height and depletion width-a “height–width" hybrid mechanism.

Supplementary Note 6: SCM-derived depletion width in the planar FTJ with p-Si electrode

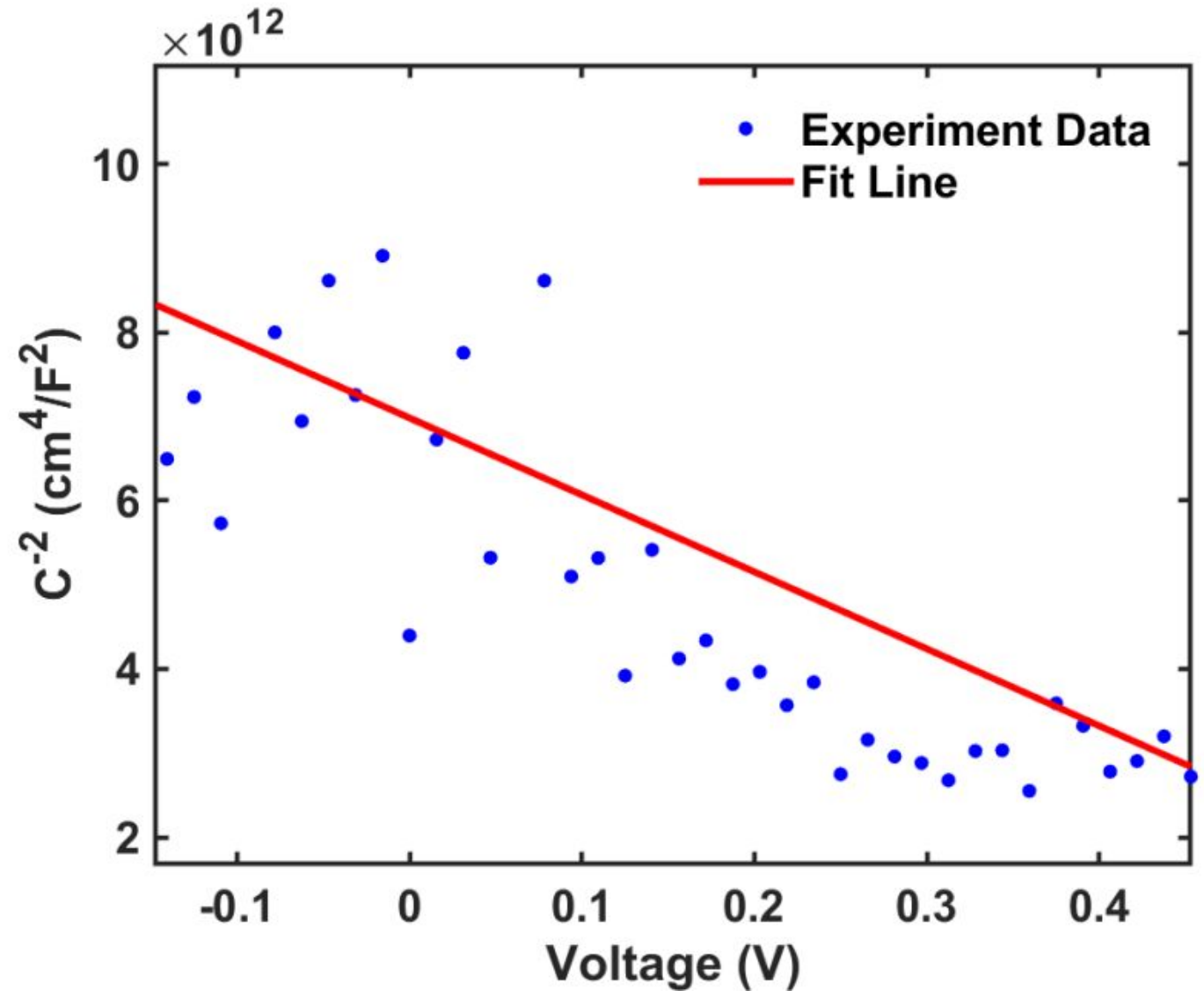

Fig. S3: SCM extraction of the depletion behavior in the planar Au/3R-MoS_2/p-Si FTJ. The OFF-state $C^{-2} - V_d$ response exhibits a linear dependence on the applied bias, consistent with depletion-type interfacial behavior. The linear fit yields a built-in potential of $V_{\mathrm{bi}} = 0.763$ V.

To demonstrate the "height–width" hybrid mechanism also exists in our planar Au/3R-MoS_2/p-Si FTJ, we performed SCM analysis on the 3R-MoS_2/p-Si interface (Fig. S3) and extracted the depletion-region parameters directly from the local $C^{-2} - V$ characteristics.

As detailed in Supplementary Note 5, the depletion width (w_{d}) at the interface within the electrode is evaluated using the depletion approximation model:

$$w_{\mathrm{d}} = \sqrt{\frac{2\varepsilon_{\mathrm{s}}(V_{\mathrm{bi}} - V_{\mathrm{d}})}{qN}},$$

with $V_{\mathrm{d}} = 0.4$ V, $\varepsilon_{\mathrm{s}} = 11.7\,\varepsilon_0$, $N = 2 \times 10^{19}$ cm^{-3}, and extracted $V_{\mathrm{bi}} = 0.763$ V (Fig. S3), the depletion width on the interface of p-silicon and MoS_2 is estimated to be $w_{\mathrm{d,off}} \approx$ 4.84 nm. This value is similar to the SCM-derived depletion width of the BDD-contacted junction, confirming that heavily doped p-Si can also provide an interfacial depletion region as the tunable tunneling width.

Supplementary Note 7: First-principles calculations for the electrostatic charge redistribution as the result of polarization switching

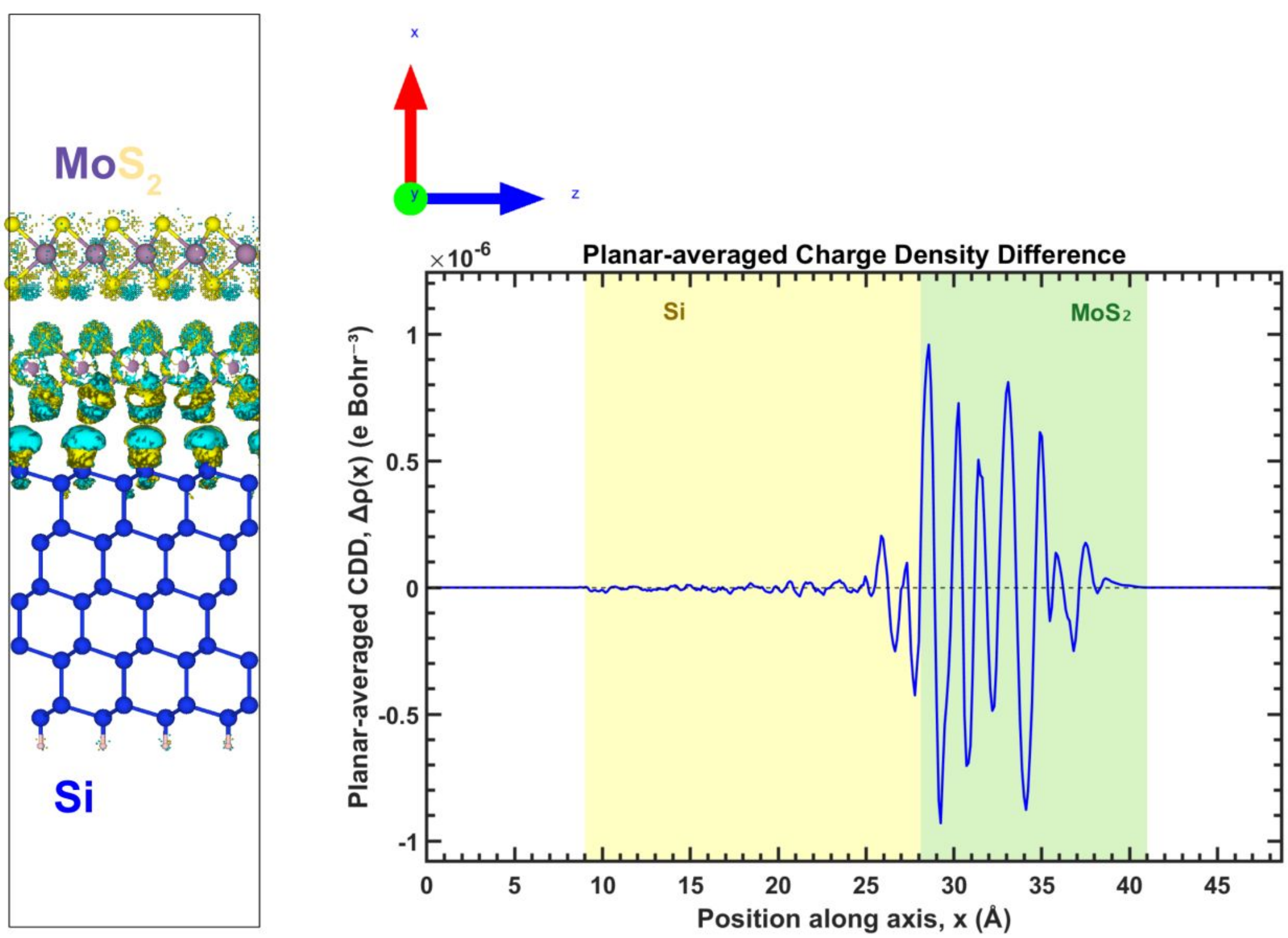

Figure S4. Polarization-dependent charge redistribution at the bilayer 3R-MoS_2/p-Si interface. Left: three-dimensional differential charge-density distribution difference

between the AB and BA polarization configurations. Yellow and cyan isosurfaces represent relative electron accumulation and depletion, respectively. Right: corresponding planar-averaged charge-density difference along the out-of-plane direction. Clear charge redistribution occurs within bilayer 3R-MoS_2 and the Si side, extending into the Si side before decaying toward the interior of the Si. Notice that we use 1 B atom in 159 Si atoms in our simulation, calculated to be approximately 3×10^{20} cm^{-3}, over 15 times higher than the experimental value of 2×10^{19} cm^{-3}, resulting in a substantially shorter extension in Si.

Calculations for the AB and BA configurations were performed using first-principles density functional theory (DFT)[4,5] as implemented in OpenMX[6–8] with the Perdew-Burke-Ernzerhof (PBE)[9] exchange-correlation energy functional in the generalized gradient approximation (GGA)_on a 342-atom structure consisting of a 5×5 bilayer 3R-MoS_2 supercell and an H-terminated Si (111) slab. Effective screening medium (ESM)[10,11] method was used for dipole correction, along with a cutoff energy of 220 Ryd, a regular k-grid mesh of 4×4×1, and the standard optimized PAO basis functions, and a 20 Å vacuum along the out-of-plane direction. The charge density was extracted and plane-averaged along the out of plane direction for analysis. The experimental p-Si doping concentration is 2×10^{19} cm^{-3}, requiring approximately one B atom for every 2,500 Si atoms, together with a Si slab sufficiently thick to accommodate the several-nanometer space-charge region. The resulting interface supercell would therefore contain several thousand atoms, which is computationally demanding for direct first-principles calculations. We therefore adopted the above smaller with one substitutional B atom among 159 Si atoms, corresponding to an effective doping concentration of approximately 3×10^{20} cm^{-3}. This model is intended to

provide qualitative insight into the local polarization-dependent interface response rather than to reproduce the experimental depletion width quantitatively.

As shown in Fig. S4, both three-dimensional differential charge-density distribution $\Delta\rho$ (left) and the planar-averaged charge-density difference (right) show clear charge redistribution within bilayer 3R-MoS_2, and on the Si surface upon switching from AB to BA stacking. Here $\Delta\rho$ was calculated as the difference between the AB and BA differential charge densities, focusing on the change in the charge density induced by the AB/BA switching. In particular, a clear polarization-dependent redistribution of charge extends from the interface into the Si substrate, indicating that reversal of the sliding polarization modifies the interfacial charge transfer and the carrier distribution on the Si side. On the Si side, the planar-averaged profile exhibits a predominantly negative charge redistribution near the interface, which confirms a net increase in negative charge in the BA state relative to the AB state. For p-type Si, such an increase in negative space charge is consistent with enhanced depletion of mobile holes. These results therefore provide microscopic evidence that reversal of the sliding polarization modifies the interfacial charge transfer and modulates the depletion condition on the Si side. We note that the several-angstrom spatial extent observed in the first-principles calculation should not be interpreted as the quantitative depletion width of the experimental device. As mentioned above, the Si doping concentration used in the DFT supercell is substantially higher than that of our experimental substrate; specifically, the experimental doping concentration is approximately 1/15 of that employed in the calculation. A lower carrier concentration is expected to result in a substantially larger depletion width in the actual device. Therefore, the qualitative first-principles calculation captures the atomic-scale origin of the polarization-dependent

interfacial charge transfer and Si-side depletion, which supports the depletion width modulation picture.

Supplementary Note 8: Sweep-rate dependence of TER and multiple planar sliding FTJs

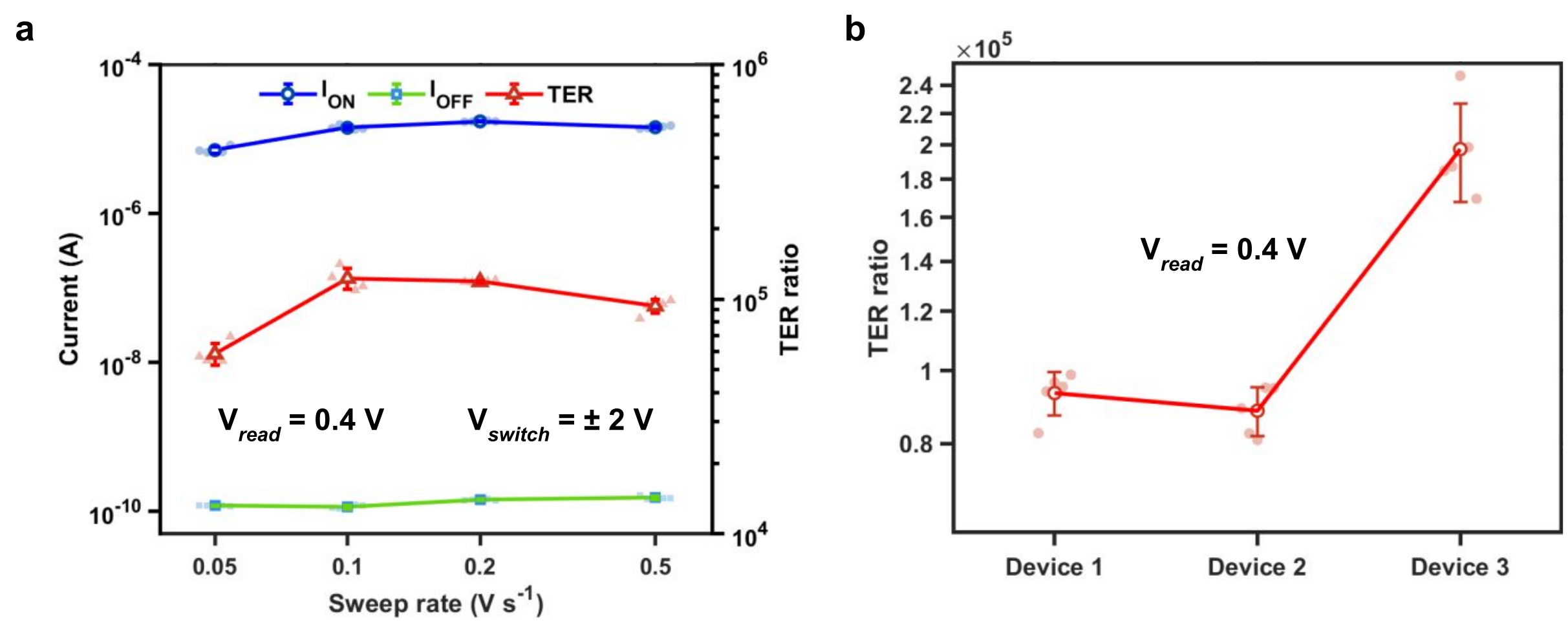

Fig. S5: Sweep-rate dependence and device-to-device reproducibility of Au/3R-MoS_2/p-Si FTJs. (a) ON-state current, OFF-state current and TER ratio extracted from I–V measurements at different voltage sweep rates. The current values were extracted at V_{read} = 0.4 V, and the TER was calculated as (I_{ON}/I_{OFF}). The switching voltage was V_{switch} = ± 2 V. Pale symbols show individual measurements, and open symbols show error bars of the device at each sweep rate. The ON/OFF currents and TER remain within the same order of magnitude and show no clear dependence on sweep rate, indicating that the giant TER is not dominated by slow ion migration or trapped charges.(b) TER ratios extracted from three independent Au/3R-MoS_2/p-Si FTJ devices at V_{read} = 0.4 V. Pale symbols show individual

measurements, and open symbols show error bars of the TER of each device. All devices show TER on the order of (10^5), confirming the device-to-device reproducibility of the planar sliding FTJs.

Hysteretic resistance switching in two-terminal FTJs may arise from non-ferroelectric mechanisms, including charge trapping at defects, ion migration, and filamentary formation.[12–14] However, these mechanisms typically produce characteristic signatures such as randomness of switching voltages in filamentary devices, as well as sweep-to-sweep drift, threshold shifts, slow relaxation, or cycling-induced fatigue in trap- or ion-mediated switching.[15–17] These features are not observed in our sliding FTJs. Instead, the devices show smooth and reproducible I–V curves, nearly overlapping switching loops, no clear current drift, and stable ON and OFF currents during cycling (Fig. 2a,b and Fig. 3e,f). In addition, an ion-migration mechanism should generally exhibit sweep-rate dependence because mobile ions or charged defects require finite time to redistribute under bias. Therefore, if slow ion motion dominated the switching, changing the voltage sweep rate would be expected to produce measurable changes in the current level, loop shape, or TER. In our planar FTJ device, the ON-state current, OFF-state current and TER ratio remain within the same order of magnitude and show no clear change on sweep rate (Fig. S5). This further excludes ion migration's role in sliding FTJs. Therefore, these observations indicate that such effects are not the dominant origin of TER at the level of 10^5 observed in our devices.

Supplementary Note 9: SS-PFM Details

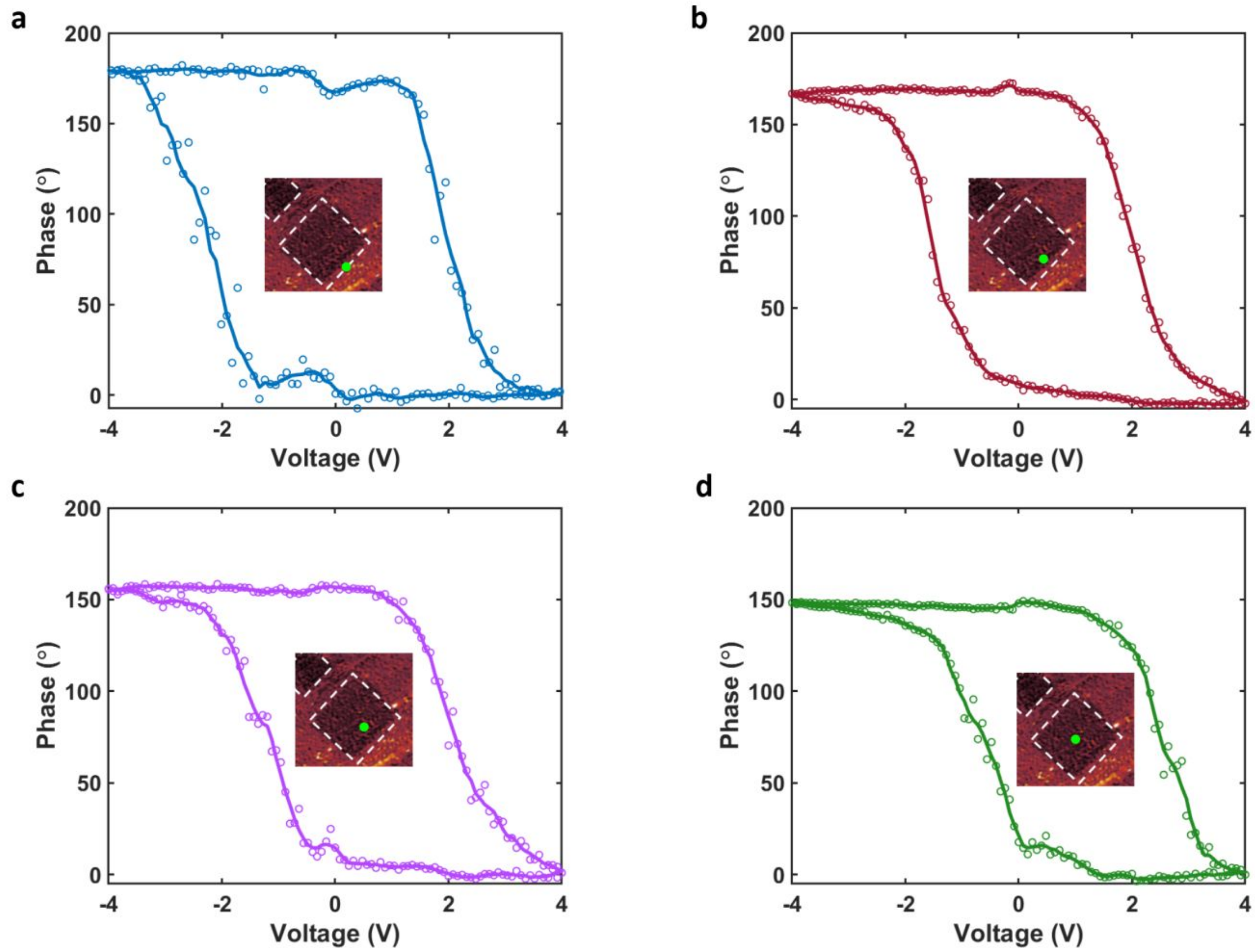

Fig. S6: SS-PFM at different domain positions. (a-d) SS-PFM measurement phase plots that were performed at different positions with varying distances relative to the ferroelectric domain wall. The data demonstrate that the switching behavior changes as the distance from the domain wall: clear 180° polarization reversal is achieved near the boundary, while the phase contrast decreases as the measurement point moves towards the center of the domain, indicating incomplete switching. The insertions show the PFM mapping with domain boundaries highlighted by white dashed lines, and the green points show where the measurements were performed.

SS-PFM was performed to analyze the local switching behavior of the phase response at selected points of the Au/3R-MoS_2/BDD FTJs, with respect to their positions relative to the domain walls. As established in previous studies,[18,19] a full 180° phase inversion in SS-PFM hysteresis loops signifies complete ferroelectric polarization reversal, whereas a reduced phase contrast indicates incomplete switching.

In the SS-PFM measurements, we observed similar complete and incomplete switching behavior. In Fig. S6 (a-d), under the same voltage sweep conditions, measurements conducted at points closer to the domain wall show a full 180° phase contrast between AB and BA domains, while points located further away from the domain wall exhibit more incomplete switching behavior, with phase contrast down to $\sim 155^\circ$. By combining the phase response polarization switching relationships from previous studies[18,19] with our measured potential difference of 130 meV between AB and BA states, we estimate that the barrier height modulation decreased by 20 meV from Fig. S6a to Fig. S6d. This behavior can be attributed to the nucleation-limited switching mechanism. The domain wall acts as a pre-existing boundary with a lower energy barrier, serving as a preferential nucleation site that facilitates polarization reversal more easily (through domain wall motion). Conversely, within the domain interior, the switching process requires nucleation of a new domain, which is energetically more demanding due to the mechanical clamping constraints and the lack of defects to aid nucleation.[18] Consequently, switching is partially suppressed away from the domain wall under the same applied bias window.

Supplementary Note 10: Theoretical Modeling of TER Variations induced by Strain and Domain Walls

Based on the tunneling transport model established in Supplementary Note 4, we further investigated the impact of strain and domain walls on the TER of the Au/3R-MoS_2/BDD FTJ. The theoretical TER is determined by the contrast in barrier height (ϕ) and total barrier width (d) between the ON and OFF states, expressed as:

$$\mathrm{TER} = \exp\left[\frac{2\sqrt{2m^*}}{\hbar}\left(\sqrt{\phi_{\mathrm{off}}}\,d_{\mathrm{off}} - \sqrt{\phi_{\mathrm{on}}}\,d_{\mathrm{on}}\right)\right],$$

where $d_{\mathrm{on}} = d_{\mathrm{FE}}$ and $d_{\mathrm{off}} = d_{\mathrm{FE}} + w_{\mathrm{d}}$. To capture the realistic behavior of the device under operating conditions, we model two scenarios that can influence the TER derived in Eq. (S4):

1. **Strain-Induced TER Modulation:** Tensile strain applied to the MoS_2 modulates the band structure and polarization. Specifically, strain leads to a reduction in the semiconductor bandgap and weakening of the ferroelectric polarization[20]. Due to such bandgap narrowing effect, 1% of tensile strain corresponds to a barrier height reduction of approximately 0.13 eV in both the On and OFF states.[21] Moreover, strain will introduce counter-polarization in a different direction and weaken the polarization about 40% under 1% strain.[22] This effect therefore reduces the depletion width modulation (Δw_d) and the effective barrier height difference ($\Delta\phi$) between ON/OFF states proportionally by the same factor of 40%, relative to the w_{d} and ϕ values used in Supplementary Note 5. As a result, the TER dropped to ~ 10, showing nearly a four-order drop.

To validate the strain-induced band gap change in bilayer 3R MoS_2, we have conducted first-principles calculations of the electronic bandgap under uniaxial tensile strain along the armchair and zigzag directions (Fig. S7). The calculations were performed using the Vienna Ab Initio Simulation Package (VASP).[23] Core electrons were treated within the projector augmented wave method,[24] and the GGA-PBE functional was used for the exchange-correlation energy. A cutoff energy of 520 eV was adopted consistently among all calculations. A 20 Å vacuum was added to the bilayer 3R-MoS_2 to minimize the image interaction due to periodic boundary condition, along with a dipole correction. To account for dispersion, Grimme's DFT-D3 method[25] was applied with zero-damping function. The initial structure and atomic coordinates were fully relaxed except for the out-of-plane *c* lattice vector which was kept fixed, until a maximal residual atomic force of < 0.001 eV $Å^{-1}$. The tensile strain was set to vary from 0% to 1.0% in increments of 0.1%. The bandgap was determined from the global valence-band maximum (VBM) and conduction-band minimum (CBM) sampled in the Brillouin zone. For self-consistent electronic structure calculation at each strain, we applied a Γ-centered k-grid sampling mesh of 15×9 ×1 for uniaxial strain along zigzag and armchair directions.

At zero strain, bilayer 3R-MoS_2 exhibits an indirect bandgap of approximately 1.279 eV. As tensile strain increases, the bandgap linearly decreases along both armchair and zigzag directions. The corresponding linear bandgap modulation rates are approximately −120.4 and −121.1 meV/% for the armchair and zigzag directions, respectively. The linear fits yield $R^2>0.999$ for both strain conditions. Throughout the investigated strain range (0-1.0%), the VBM remains located at the Γ point while the CBM remains at the K point. This value agrees with the 120 meV/% bandgap-strain relation we used in the above

section, supporting our interpretation of strain-induced narrowing of the MoS_2 bandgap reduces the effective tunneling barrier in Fig.4.

To experimentally examine the strain dependence of polarization, we performed spatially correlated Raman and KPFM measurements on the same bilayer 3R-MoS_2 flake, as shown in Fig. S8. Raman mapping was used to determine the local strain distribution, while KPFM was used to probe the corresponding polarization-related surface-potential contrast, following the same approaches employed in Fig. 4a,c and Fig. 2c. As shown in Fig. S8a, we identify two representative regions exhibiting reduced KPFM potential contrast relative to nearby regions with the maximum polarization-related potential, as highlighted by the black dashed lines. The corresponding KPFM line profiles reveal clear reductions in surface potential: Line Cut 1 shows a potential decrease of approximately 0.042 V, while Line Cut 2 shows a decrease of approximately 0.023 V. Using the relation $\Delta P = \frac{\epsilon_0 \epsilon_r (V_{brignt} - V_{dark})}{2d}$, these potential differences correspond to reductions in the effective polarization of approximately 30% in Area 1 and 15% in Area 2 relative to the region with the maximum polarization-related potential. Spatially correlated Raman mapping indicates tensile strains of approximately 0.7% and 0.34% in Areas 1 and 2, respectively. By correlating the local strain with the corresponding reduction in polarization-related potential, we obtain a strain-dependent decrease in polarization of approximately 42% per % strain, similar with the 40% per % strain trend we estimate in the section above. These spatially correlated measurements therefore provide experimental evidence that tensile strain is associated with a reduction in the polarization in bilayer 3R-MoS_2, also consistent with a recent theoretical study reporting reduced polarization under tensile strain induced by lattice modification.[26]

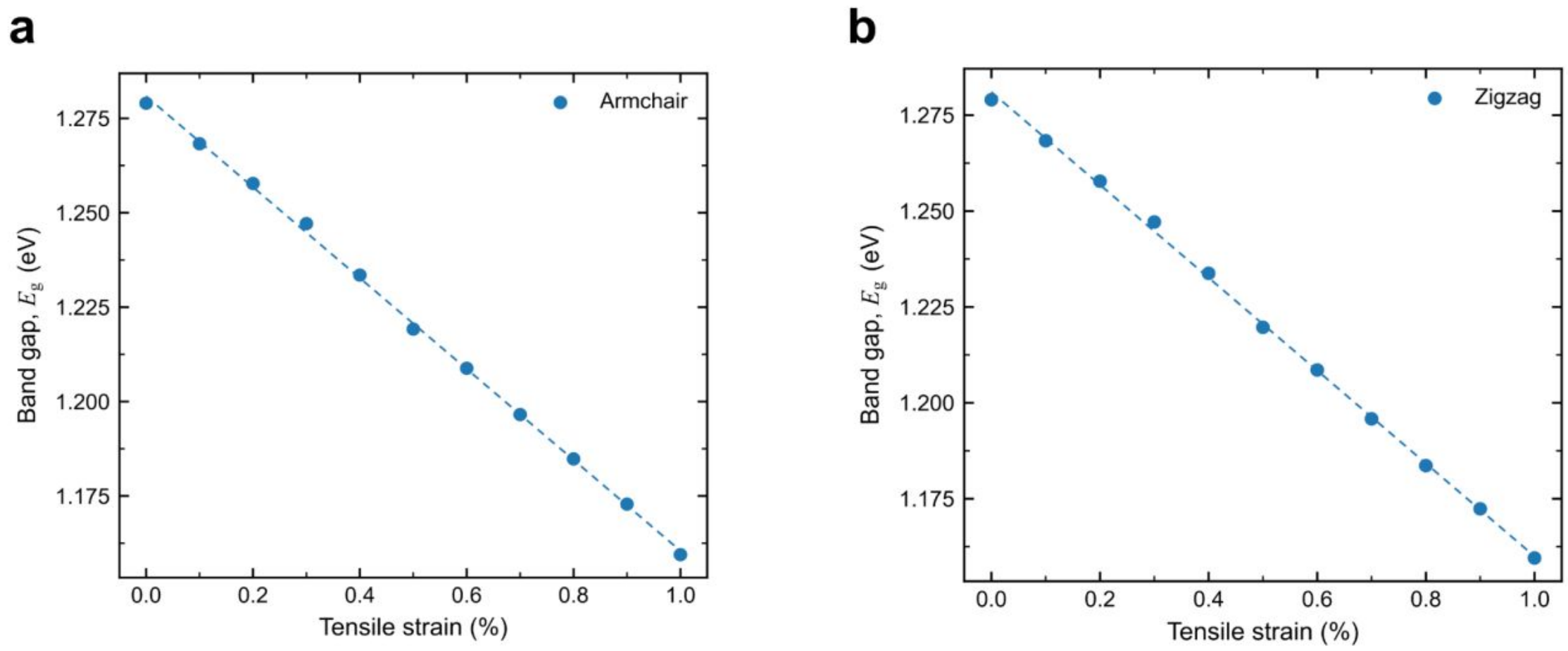

Fig. S7: Strain-dependent electronic bandgap of bilayer 3R-MoS_2. Calculated bandgap as a function of uniaxial tensile strain along (a), the armchair direction; (b), the zigzag direction. Tensile strain was varied from 0% to 1.0% in increments of 0.1%. Symbols (circles) represent the DFT-calculated values, and dashed lines show linear fits. In both cases, the bandgap decreases nearly linearly with increasing tensile strain.

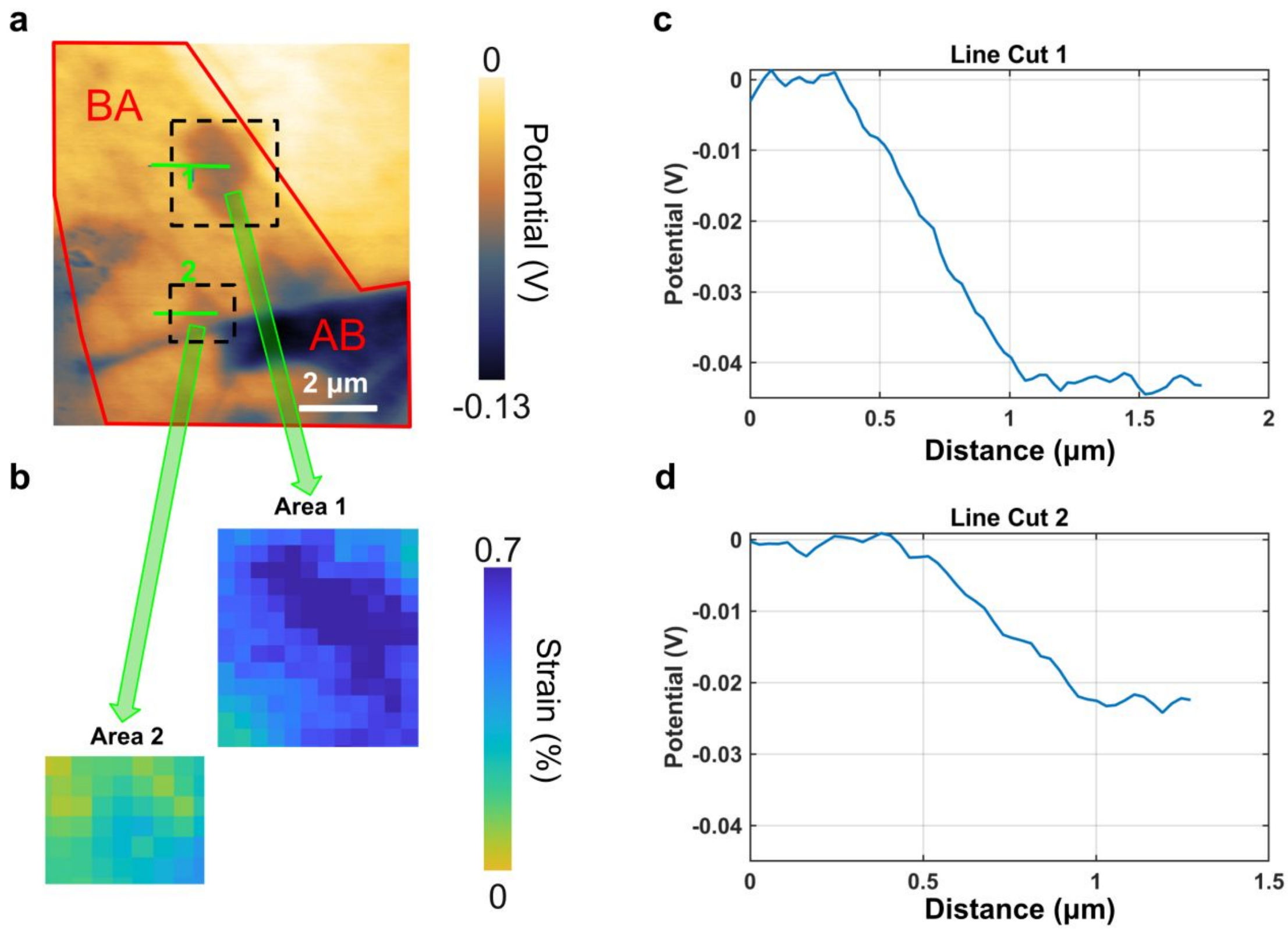

Fig. S8: Spatial correlation between local tensile strain and polarization-related surface potential in bilayer 3R-MoS_2. (a) KPFM surface-potential map of a bilayer 3R-MoS_2 flake, where bright area is BA stacking and black area is AB stacking. In the BA stacking area, some dimmer parts are strained BA stacking areas, such as area 1 and area 2. The red line outlines the bilayer flake boundary, and the green lines indicate the positions of Line Cuts 1 and 2, which cross the strain-free and strained area in BA stacking. (b) Raman-derived tensile-strain map acquired from area 1 and area 2 in **a**, with 0.7% strain and 0.34% strain, respectively. (c, d) KPFM potential profiles along Line Cuts 1 and 2, showing potential decreases of approximately 0.042 and 0.023 V, respectively, corresponding to polarization decreases by 30% and 15%.

2. **Incomplete Switching Related to Domain Wall:** The degree of ferroelectric polarization switching is spatially dependent. Regions close to domain walls exhibit complete polarization reversal with 180° phase contrast (Fig. S6), maximizing the TER. Conversely, regions deeper into the domain center (farther from the domain wall) suffer from incomplete switching due to domain pinning or insufficient electric field, leading to a reduced effective barrier height and thinner depletion width in the OFF state.[27,28] Based on the SS-PFM results in Supplementary Note 9, the SS-PFM phase plot indicates the polarization decreased by 15% over a distance of $2\ \mu\text{m}$, therefore 20 meV decrease in barrier height modulation. Accordingly, both the $\Delta\phi$ and Δw_d decrease proportionally with the same fraction, therefore leading to a one-order decrease in TER from 3×10^5 to 3×10^4.

Based on the models in the two scenarios above, we calculated TER as a function of both strain and distance to the domain wall, and the fit curves are shown in Fig. 4e and Fig. 4f. This simulation reveals that the TER is exponentially influenced by both strain and distance to the domain wall, but is more sensitive to strain than to domain wall distance. The theoretical model enhances the evidence that the giant TER originates from the "height-width" hybrid mechanism and reveals that the giant TER ($> 10^6$) can be well preserved in the regions of low strain and close to the domain wall.

Supplementary Note 11: Raman Spectroscopy of Strain-free and Strained Regions

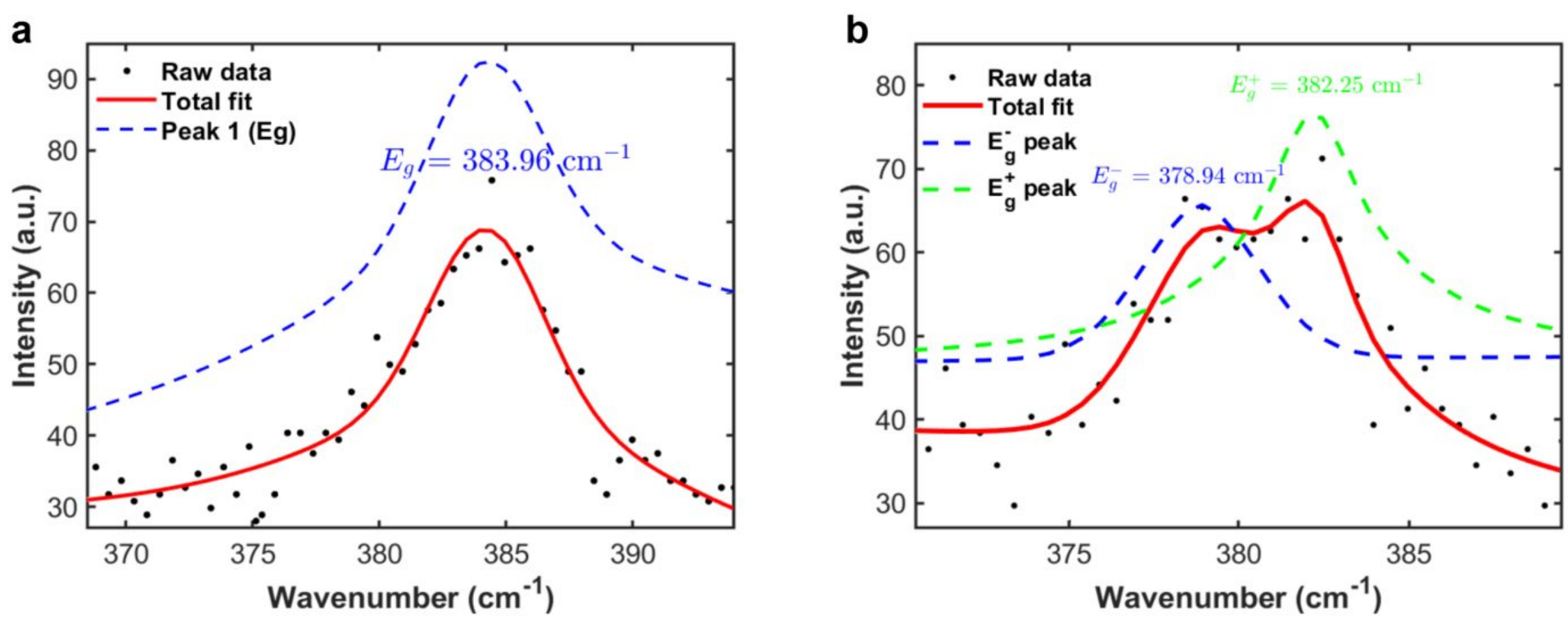

Fig. S9: Raman Spectroscopy of Strain-free and Strained Regions. (a-b) Raman spectrum measured on the strain-free region and the strained region of the 3R MoS_2 . In the strain-free region, the spectrum shows a single E_g peak centered at 383.96 cm^{-1} without any splitting or negligible shifting. In the strained region, the peak exhibits clear splitting and the E_g^- mode shifts to 378.94 cm^{-1}, corresponding to a significant shift of 5.06 cm^{-1} relative to the strain-free case.

Raman spectroscopy was employed to probe the local strain distribution and quantify the magnitude of the strain on the bilayer 3R MoS_2. According to previous studies, the in-plane vibrational mode (E_g) of unstrained bilayer MoS_2 is typically centered around ~ 384 cm $^{-1}$.[21,29] Theoretically, the threefold rotational symmetry (D_{3h}) of the lattice is broken under strain. This symmetry breaking lifts the degeneracy of the E_g mode, causing it to split into two sub-peaks E_g^+ (with a lower shift rate) and E_g^- (with a higher shift rate).

Guided by these principles, we analyzed the collected spectra. As shown in Fig. S9a, the spectrum from specific regions exhibits a single peak centered at 383.96 cm^{-1}. Since this value closely aligns with the standard unstrained case, we identify these regions as strain-free. In contrast, a typical spectrum obtained from the strained region (Fig. S9b) reveals a distinct splitting of the peak into a lower-frequency branch (E_g^- at 378.94 cm^{-1}) and a higher-frequency branch (E_g^+ at 382.25 cm^{-1}).

To quantify the local strain, we utilized the frequency shift of the highly strain-sensitive E_g^- branch. Based on established strain shift models for bilayer MoS_2, the E_g^- mode exhibits a shift rate of approximately -4.0 cm^{-1} per 1% of uniaxial strain[29]. For example, the observed shift of ~ 5.06 cm^{-1} (relative to the pristine peak position of 384 cm^{-1}) corresponds to a local tensile strain of approximately 1.2%. This quantitative analysis provides a basis for the subsequent investigation of the strain-TER relationship in Fig. 4.

Supplementary Note 12: EFM and Raman Characterization of the Au/3R-MoS$_2$/p-Si FTJ

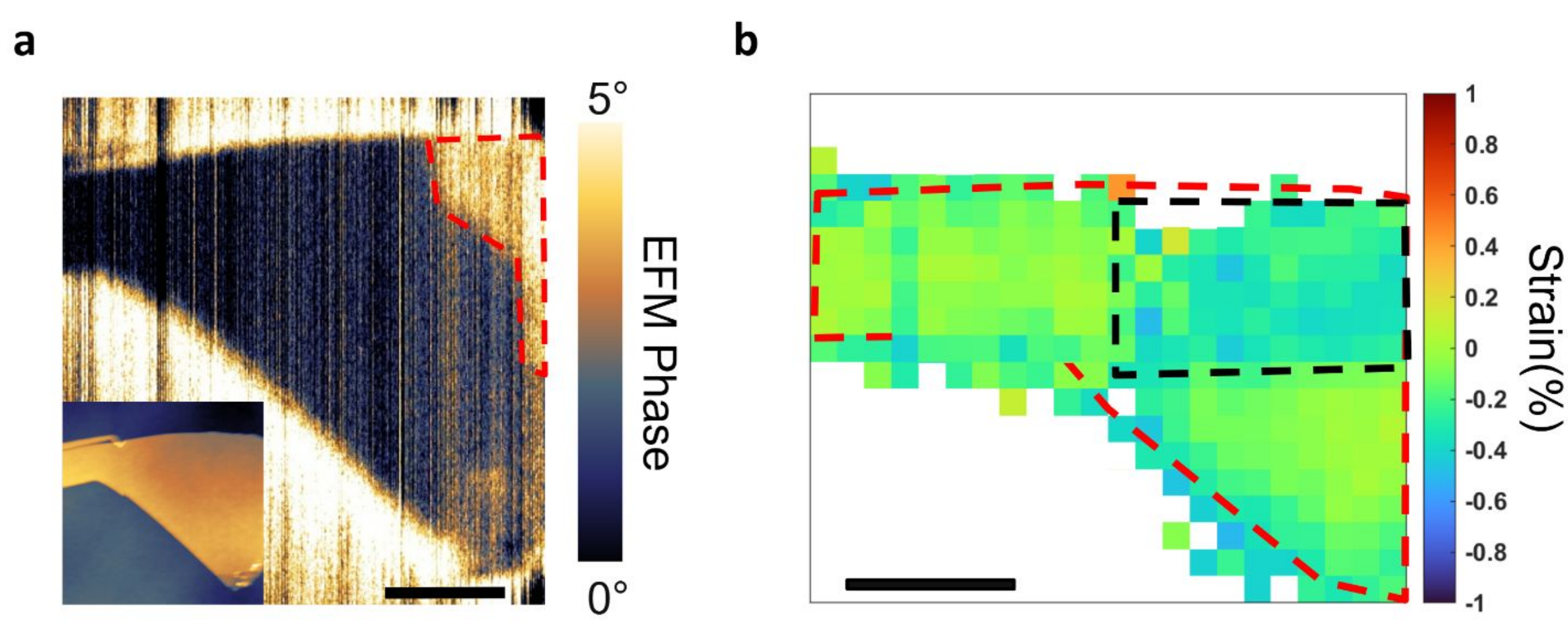

Fig. S10: Spatial characterization of the Au/3R-MoS_2/p-Si FTJ. (a) EFM phase image of the bilayer 3R-MoS_2 flake before transferring onto the p-Si substrate. The phase contrast reveals pre-existing domains with opposite out-of-plane polarizations and a clear domain wall, marked by the red dashed lines. The inset displays the corresponding AFM topography of the 3R-MoS_2 flake. Scale bar: 2 μm. (b) Spatial strain mapping of the same MoS_2 flake derived from Raman spectroscopy. The red dashed line outlines the 3R-MoS_2 flake, while the black dashed box indicates the position of the top Au electrode. The active junction area exhibits minimal compressive strain below 0.2%, ensuring the device maintains an ultrahigh TER. Scale bars: 2 μm.

As demonstrated in the main text, the choice of BDD or p-Si electrode can significantly boost the TER by employing the hybrid height-width modulation. To validate these design principles in a scalable FTJ device, we performed detailed spatial characterizations on the planar Au/3R-MoS_2/p-Si FTJ device and exclude the strain's impact to maximize the performance.

Before picking up the bilayer 3R-MoS_2 flake, Electrostatic Force Microscopy (EFM) was utilized to pre-screen the ferroelectric domains of the mechanically exfoliated bilayer 3R-MoS_2. As shown in Fig. S10a, the EFM phase image clearly resolves pre-existing ferroelectric domains separated by a distinct domain wall. Guided by our observation that polarization reversal is more complete and yields a higher TER near domain boundaries, the top Au electrode was strategically patterned near the domain wall.

More critically, because residual tensile strain was shown to drastically increase the OFF-state current and suppress the TER ratio, we ensured that the strain in the active device area is negligible. Using the same spatially resolved Raman spectroscopy method we used in Fig. 4 and Fig. S9, the extracted spatial strain mapping across the MoS_2 flake is shown in Fig. S10b (outlined by the red dashed line). Most of the bilayer 3R-MoS_2 region exhibits negligible strain, and the active junction area beneath the top Au electrode (marked by the black dashed box) exhibits a very small compressive strain of less than 0.2%, ensuring that the device performance is not impacted by local strain. By simultaneously minimizing residual strain and utilizing the hybrid modulation, this optimized device architecture successfully preserves the ultrahigh TER exceeding 10^5.

Supplementary Note 13: Temperature-dependent tunneling characterization of the FTJs

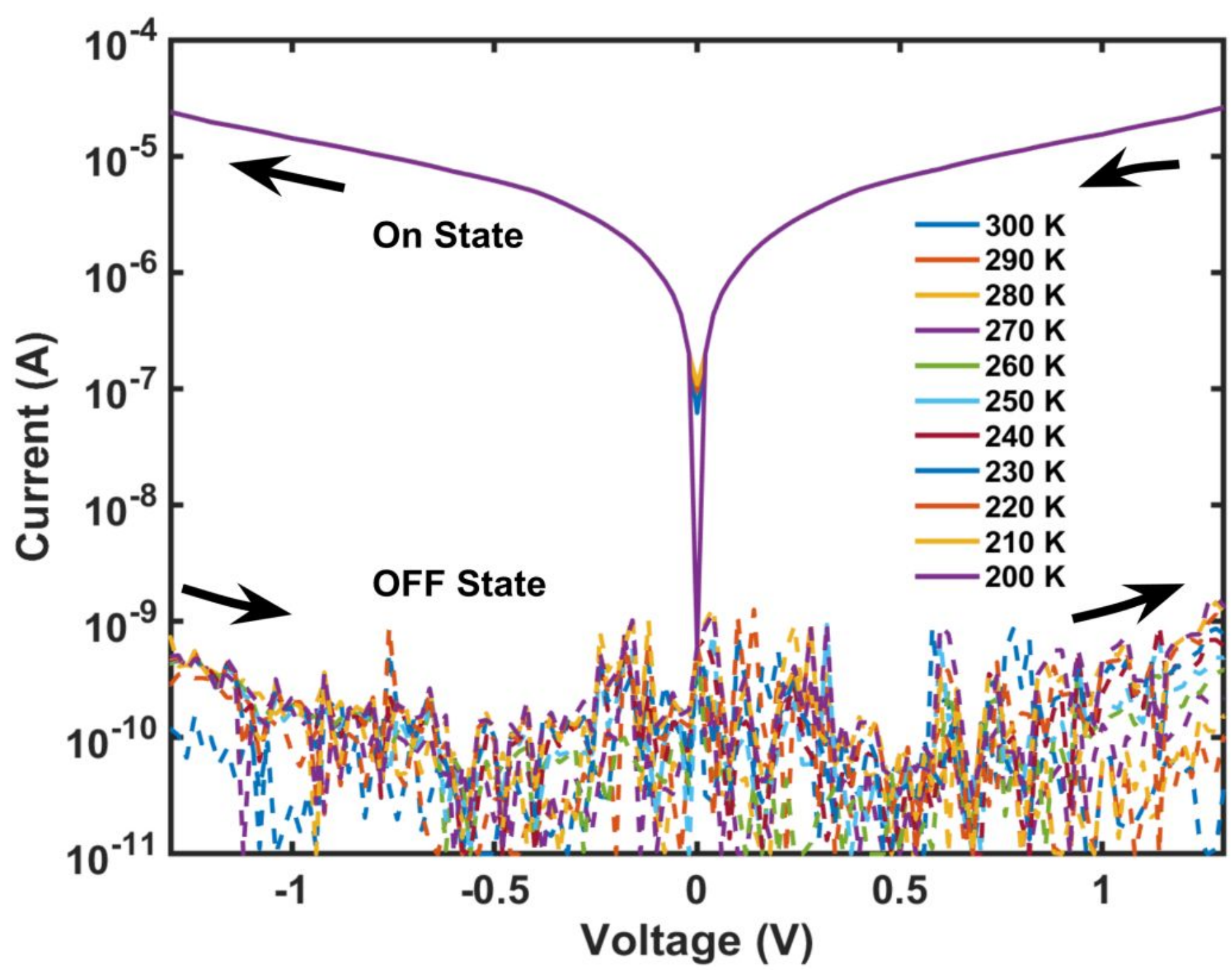

Fig. S11: Temperature-dependent current-voltage curves of bilayer 3R-MoS_2-based planar FTJ. ON- and OFF-state current–voltage characteristics measured from 200 to 300 K. The ON-state currents (solid lines), shown by the upper group of curves, nearly overlap over the measured temperature range. The OFF-state currents (dashed lines) remain in the range of approximately 10^{-11}~10^{-10} A and are comparable to the instrumental noise floor. This shows that the TER is robust over a large T range. The black arrows indicate the sweep directions.

For an ideal quantum tunneling junction, the current should generally show weak temperature dependence, as a carrier tunnels through the barrier rather than acquiring thermal energy to go over it. To reveal temperature's role in the sliding FTJ, we fabricated a new bilayer 3R-MoS_2-based planar ferroelectric tunnel junction, the same as the one shown in Fig. 3e. The tunneling behavior was characterized in a Physical Property Measurement System with controlled temperatures.

We measured temperature-dependent current–voltage characteristics from 200 to 300 K in 10 K increments and compared the ON- and OFF-state currents within the read-voltage range of -1.3 V to +1.3 V. As shown in Fig. S11, the ON-state I-V curves nearly overlap over the entire temperature range. Thermally activated transport, such as thermionic emission, is expected to increase strongly with temperature, whereas direct tunneling generally exhibits only a weak temperature dependence. The absence of change in the ON-state current from 200 to 300 K is therefore consistent with tunneling-dominated transport rather than a thermo-assisted process within the investigated read-voltage range. The OFF-state current fluctuates between approximately 10^{-11}~10^{-10} A and approaches the

instrumental noise floor of 10^{-11} A. Therefore, the intrinsic temperature dependence of the OFF-state transport cannot be reliably extracted, even though the larger effective barrier width may increase the contribution of thermally assisted transport by suppressing direct tunneling,

Taken together, the temperature-dependent measurements show very weak temperature dependence, which means the high TER stays robust over the investigated temperature range.

References

1. Melitz, W.; Shen, J.; Kummel, A. C.; Lee, S. Kelvin Probe Force Microscopy and Its Application. *Surf. Sci. Rep.* **2011**, *66*, 1–27.
2. Gruverman, A.; Wu, D.; Lu, H.; Wang, Y.; Jang, H. W.; Folkman, C. M.; Zhuravlev, M. Ye.; Felker, D.; Rzchowski, M.; Eom, C.-B.; Tsymbal, E. Y. Tunneling Electroresistance Effect in Ferroelectric Tunnel Junctions at the Nanoscale. *Nano Lett.* **2009**, *9*, 3539–3543.
3. Xi, Z.; Ruan, J.; Li, C.; Zheng, C.; Wen, Z.; Dai, J.; Li, A.; Wu, D. Giant Tunnelling Electroresistance in Metal/Ferroelectric/Semiconductor Tunnel Junctions by Engineering the Schottky Barrier. *Nat. Commun.* **2017**, *8*, 15217.
4. Kohn, W.; Sham, L. J. Self-Consistent Equations Including Exchange and Correlation Effects. *Phys. Rev.* **1965**, *140*, A1133–A1138.
5. Hohenberg, P.; Kohn, W. Inhomogeneous Electron Gas. *Phys. Rev.* **1964**, *136*, B864–B871.
6. Ozaki, T.; Kino, H. Numerical Atomic Basis Orbitals from H to Kr. *Phys. Rev. B* **2004**, *69*, 195113.
7. Ozaki, T.; Kino, H. Efficient Projector Expansion for the *ab initio* LCAO Method. *Phys. Rev. B* **2005**, *72*, 045121.
8. Ozaki, T. Variationally Optimized Atomic Orbitals for Large-Scale Electronic Structures. *Phys. Rev. B* **2003**, *67*, 155108.
9. Perdew, J. P.; Burke, K.; Ernzerhof, M. Generalized Gradient Approximation Made Simple. *Phys. Rev. Lett.* **1996**, *77*, 3865–3868.

10. Ohwaki, T.; Otani, M.; Ikeshoji, T.; Ozaki, T. Large-Scale First-Principles Molecular Dynamics for Electrochemical Systems with O(N) Methods. *J. Chem. Phys.* **2012**, *136*, 134101.

11. Otani, M.; Sugino, O. First-Principles Calculations of Charged Surfaces and Interfaces: A Plane-Wave Nonrepeated Slab Approach. *Phys. Rev. B* **2006**, *73*, 115407.

12. Waser, R.; Aono, M. Nanoionics-Based Resistive Switching Memories. *Nat. Mater.* **2007**, *6*, 833–840.

13. Park, J.; Kumar, A.; Zhou, Y.; Oh, S.; Kim, J.-H.; Shi, Y.; Jain, S.; Hota, G.; Qiu, E.; Nagle, A. L.; Schuller, I. K.; Schuman, C. D.; Cauwenberghs, G.; Kuzum, D. Multi-Level, Forming and Filament Free, Bulk Switching Trilayer RRAM for Neuromorphic Computing at the Edge. *Nat. Commun.* **2024**, *15*, 3492.

14. Sun, W.; Gao, B.; Chi, M.; Xia, Q.; Yang, J. J.; Qian, H.; Wu, H. Understanding Memristive Switching *via in situ* Characterization and Device Modeling. *Nat. Commun.* **2019**, *10*, 3453.

15. Jeong, D. S.; Schroeder, H.; Breuer, U.; Waser, R. Characteristic Electroforming Behavior in Pt/TiO_2/Pt Resistive Switching Cells Depending on Atmosphere. *J. Appl. Phys.* **2008**, *104*, 123716.

16. Yang, Y.; Wu, M.; Zheng, X.; Zheng, C.; Xu, J.; Xu, Z.; Li, X.; Lou, X.; Wu, D.; Liu, X.; Pennycook, S. J.; Wen, Z. Atomic-Scale Fatigue Mechanism of Ferroelectric Tunnel Junctions. *Sci. Adv.* **2021**, *7*, eabh2716.

17. Yang, Y.; Gao, P.; Gaba, S.; Chang, T.; Pan, X.; Lu, W. Observation of Conducting Filament Growth in Nanoscale Resistive Memories. *Nat. Commun.* **2012**, *3*, 732.

18. Jesse, S.; Baddorf, A. P.; Kalinin, S. V. Switching Spectroscopy Piezoresponse Force Microscopy of Ferroelectric Materials. *Appl. Phys. Lett.* **2006**, *88*, 062908.

19. Soergel, E. Piezoresponse Force Microscopy (PFM). *J. Phys. D: Appl. Phys.* **2011**, *44*, 464003.

20. Bhattacharyya, S.; Pandey, T.; Singh, A. K. Effect of Strain on Electronic and Thermoelectric Properties of Few Layers to Bulk MoS_2. *Nanotechnology* **2014**, *25*, 465701.

21. Conley, H. J.; Wang, B.; Ziegler, J. I.; Haglund, R. F., Jr.; Pantelides, S. T.; Bolotin, K. I. Bandgap Engineering of Strained Monolayer and Bilayer MoS_2. *Nano Lett.* **2013**, *13*, 3626–3630.

22. Duerloo, K.-A. N.; Ong, M. T.; Reed, E. J. Intrinsic Piezoelectricity in Two-Dimensional Materials. *J. Phys. Chem. Lett.* **2012**, *3*, 2871–2876.

23. Kresse, G.; Furthmüller, J. Efficient Iterative Schemes for *ab initio* Total-Energy Calculations Using a Plane-Wave Basis Set. *Phys. Rev. B* **1996**, *54*, 11169–11186.

24. Blöchl, P. E. Projector Augmented-Wave Method. *Phys. Rev. B* **1994**, *50*, 17953–17979.

25. Grimme, S.; Antony, J.; Ehrlich, S.; Krieg, H. A Consistent and Accurate *ab initio* Parametrization of Density Functional Dispersion Correction (DFT-D) for the 94 Elements H-Pu. *J. Chem. Phys.* **2010**, *132*, 154104.

26. Mu, W.; Ke, C.; Guo, N.; Zhang, Z.; Yang, L.; Li, Z.; Zhou, Y.; Li, J.; Yue, S.; Zheng, J.; Zheng, Q.; Liu, S.; Jiao, L. Sliding Ferroelectricity in MoS_2/ReS_2 Heterojunction for a Sensitive and Reconfigurable Bioelectronic Interface. *ACS Nano* **2026**, *20*, 13758–13766.

27. Liang, J.; Yang, D.; Xiao, Y.; Chen, S.; Dadap, J. I.; Rottler, J.; Ye, Z. Shear Strain-Induced Two-Dimensional Slip Avalanches in Rhombohedral MoS_2. *Nano Lett.* **2023**, *23*, 7228–7235.

28. Yang, D.; Liang, J.; Wu, J.; Xiao, Y.; Dadap, J. I.; Watanabe, K.; Taniguchi, T.; Ye, Z. Non-Volatile Electrical Polarization Switching *via* Domain Wall Release in 3R-MoS_2 Bilayer. *Nat. Commun.* **2024**, *15*, 1389.

29. Lee, J.-U.; Woo, S.; Park, J.; Park, H. C.; Son, Y.-W.; Cheong, H. Strain-Shear Coupling in Bilayer MoS_2. *Nat. Commun.* **2017**, *8*, 1370.
